# Superionic states formation in group III oxides irradiated with ultrafast lasers

**DOI:** 10.1038/s41598-022-09681-0

**Published:** 2022-04-05

**Authors:** R. A. Voronkov, N. Medvedev, A. E. Volkov

**Affiliations:** 1 P. N. Lebedev Physical Institute of the Russian Academy of Sciences, Leninskij pr., 53, 119991 Moscow, Russia; 2 Institute of Physics, Czech Academy of Sciences, Na Slovance 2, 182 21 Prague 8, Czech Republic; 3 Institute of Plasma Physics, Czech Academy of Sciences, Za Slovankou 3, 182 00 Prague 8, Czech Republic

**Keywords:** Phase transitions and critical phenomena, Structure of solids and liquids, Materials chemistry, Theory and computation

## Abstract

After ultrafast laser irradiation, a target enters a poorly explored regime where physics of a solid state overlaps with plasma physics and chemistry, creating an unusual synergy—a warm dense matter state (WDM). We study theoretically the WDM kinetics and chemistry in a number of group III-metal oxides with highly excited electronic system. We employ density functional theory to investigate a possibility of nonthermal transition of the materials into a superionic state under these conditions. Atomic and electronic properties of the materials are analyzed during the transitions to acquire insights into physical mechanisms guiding such transformations.

## Introduction

A high-intensity femtosecond laser irradiation of solids excites an electronic system of a material, whereas an ionic system is initially unaffected. Two main mechanisms govern structure transformations in materials after the laser pulses^1^. A thermal one is initiated via electron–ion (electron–phonon) coupling equilibrating a highly excited electronic system with the ionic one^2^. This coupling results in a noticeable lattice heating when an amount of energy transferred from electrons to atoms becomes significant, typically at timespans > 1 ps^2,3^.

The second one is a nonthermal channel, which may occur at much shorter times^4–7^. Nonthermal mechanism is induced by a significant energy density deposited into the electronic system, usually corresponding to the electronic temperature of a few eV^8^. Such excitation of the electronic system temporarily changes a potential energy surface of the atomic system in a solid. Appearing uncompensated forces initiate movements of atoms trying to find their new equilibrium positions and can even cause nonthermal structure transitions. In some materials, depending on the excitation level, these new equilibrium positions may not exist, and nonthermal melting ensues even when the atomic temperature does not exceed the melting point of the material^6,7,9,10^. Nonthermal atomic movement dominates at timescales under 0.5–1 ps, until the thermal channel starts to play a significant role^1^.

Materials under such conditions are one of the types of manifestations of the warm dense matter state (WDM)—an intermediate state between a cold solid and a hot plasma^11^.

It may lead to exotic structures and phases (usually transient), that are not achievable at equilibrium conditions^8,12^. One of such unusual phases is the superionic one^13^. It consists of one subsystem of a compound in a liquid state, whereas the other one is in a solid phase, simultaneously. In conventional superionic materials such as metal-lithium compounds, the high ionic diffusion conductivity is a result of sparse atomic structure^14^. In contrast, under extreme conditions, an entire sublattice may be destabilized exhibiting a liquid behavior. Such superionic state was recently produced by laser-induced dynamical shock compression of water ice^15^. Presumably, a similar superionic ice can be found naturally in giant planets interiors such as Uranus, Neptune, or other exoplanets^16^.

Superionic alumina was predicted to form after fs-laser irradiation^12^. This stable during hundreds of femtoseconds state with liquid oxygen sublattice and solid aluminum one may be produced in irradiation spots of free-electron lasers^17–19^.

However, general mechanisms leading to superionic state formation in materials were not discussed in Ref.^12^ because only α-Al_2_O_3_ (corundum) was investigated. Understanding the formation mechanism of such states should inspire experiments and theoretical research aimed at production of new superionic materials including those under less extreme conditions. It may open up opportunities for potential applications in areas requiring materials with controlled atomic ordering.

In this paper, we study a possibility of nonthermal transitions in a few oxides formed by group III metal: Al_2_O_3_, Ga_2_O_3_, In_2_O_3_, and In_2_S_3_ for comparison, to gather some statistics of materials demonstrating superionic behavior under extreme electronic excitation. These materials also have the same R-3c group symmetry as that of α-Al_2_O_3_ and their electronic structure is formed by overlap of *ns*^2^*np*^1^ and *ms*^2^*mp*^4^ atomic orbitals. Additionally, we check polymorphs of these materials for the existence of a superionic state. By analyzing such material properties as group symmetry, chemical composition and electronic energy levels, we investigate which of the parameters may be driving forces of the transition into the superionic state.

We identify thresholds of electronic excitation required to produce the superionic states in some of these compounds. These thresholds should be reasonable reference points for future experiments. We also note that superionic behavior in some materials may realize as a transitional state to a stable phase, which will form at much longer timescales than those investigated in this paper.

## Methods

For all presented simulations, we use the density functional molecular dynamics (DFT-MD) simulation within the Quantum Espresso (QE) simulation package^20^. We use norm-conserving pseudopotentials from the QE library and Perdew–Burke–Ernzerhof (PBE) exchange–correlation functional^21^. Although PBE functional does not contain explicit dependence on the electronic temperature (only in terms of occupation numbers for electronic density calculation) we use it since only a few developed *T*_*e*_-dependent functionals^22,23^ were applied mostly for metals and their capability to reproduce correct band gaps is unclear. Non-hybrid functionals are known to underestimate the band gap value at ambient conditions, however, in a case of high electronic temperatures they perform much better^24^. To validate our application of the PBE functional, we performed a cross-checking simulation for one material with—one of the most advanced and modern R2SCAN meta-GGA functional^25^.

To study atomic and electronic structure dynamics during nonthermal transitions, we implement the following algorithm. After the standard procedure of a geometry optimization, the lattice is allowed to thermalize at the room temperature *T*_*i*_ = 300 K via DFT-MD with unperturbed electronic system (*T*_*e*_ = 300 K set via Fermi–Dirac smearing) during 500 fs with 0.5 fs time step. After that, we instantly elevate electronic temperature to a certain value assuming that electrons adhere to the Fermi–Dirac distribution. At this step, next DFT self-consistent cycle automatically adjusts atomic forces and electronic band structure in accordance with new electronic temperature. Then we proceed DFT-MD simulation within 1000 fs with 0.5 fs time step.

Within this procedure, we assume that electron cascades after laser irradiation can be neglected since they take a few femtoseconds in a typical FEL spot except for hard X-rays and does not significantly affect lattice dynamics^26^. This allows us to apply a thermalized distribution for the electronic ensemble at all times avoiding complex consideration of a short-living nonequilibrium stage that cannot be treated in DFT.

We further assume that the electronic temperature can stay constant throughout the simulation since electron energy loss via kinetic energy transfer to the lattice or via spatial dissipation is minor within ~ 500 fs in the central area of an irradiated volume. These assumptions are supported by the nonadiabatic tight-binding MD simulations in Al_2_O_3_ that demonstrated electron-lattice equilibration due to electron–ion coupling requires at least a few picoseconds^1^. We note, however, that all simulations beyond 500 fs in the presented work are performed only in order to confirm nonthermal transition behavior when it is unclear at shorter timescales for some compounds investigated here.

We do not consider excitonic effects as well, since we assume that a significant level of electronic excitation and induced atomic perturbations studied here do not allow excitons to form.

An NVT-ensemble (constant number of particles, volume and temperature) is used for the electronic system and an NVE-ensemble (constant number of particles, volume and energy) for the atomic system. This choice corresponds to the conditions in a bulk achieved after irradiation with an FEL pulse, where the unperturbed media maintains a constant volume of the target’s excited part for times sufficiently longer than those modeled here^27^.

Apart from the DFT-MD simulations, further analyses were performed. In order to identify affiliation of obtained energy levels to atomic orbitals, a dependence of gamma-point energy levels on the interatomic distance was constructed via Parrinello-Raman variable-cell molecular dynamics^28^ with a target pressure *P*_target_ = − 600 kbar and atoms kept in the ideal lattice positions. For this analysis, the initial state of a material was set to its ambient structure and zero atomic and electronic temperatures (the latter was set via Fermi–Dirac distribution at *T*_e_ ≈ 30 K). At each molecular dynamics step, energy levels were extracted and shifted to zero chemical potential (Fermi level). At the last step, the gamma-point projected electronic density of states (PDOS) was calculated.

Atomic potential energy surfaces were constructed in a series of calculations. For example, for one Ga or one O atom (in a fixed lattice of all other atoms) a uniform 3-dimentional grid was set on Cartesian coordinates from (*a*_*0*_ − 1) Å to (*a*_*0*_ + 1) Å, where *a*_*0*_ is a coordinate (*x*,*y* or *z*) of the equilibrium position, with 0.1 Å step. For each point of the grid, a self-consistent calculation was carried out. Then, the total energy of the electronic system representing atomic potential energy was calculated (excluding pseudo-nuclei repulsion term that becomes noticeable only at much shorter interatomic distances than those considered here).

Energy cutoff parameter controlling the size of the plane wave basis in DFT simulations was set to *E*_*cut*_ ≈ 952 eV (70 Ry) for nonthermal transitions simulations, potential energy surfaces construction and PDOS calculations for materials at ambient conditions. For energy levels of the expanding cell and corresponding PDOS calculations, the parameter was set to *E*_*cut*_ ≈ 1360 eV (100 Ry) since increasing interatomic distance requires more plane waves in the DFT basis set to describe electronic states becoming more localized around nuclei. For all calculations, supercells consisting of 80 atoms were used.

## Results

### Atomic properties

Calculated thresholds for nonthermal phase transitions in studied materials are presented in Table 1. A threshold of the electronic temperature triggering nonthermal transition and its equivalent in a number of valence electrons excited to the conduction band, as well as the initial structure and a transition (superionic or melting) are shown there for each material.

**Table 1:** Threshold parameters triggering nonthermal transitions in various materials in various phases. Here, *T*_*e*_ is the electronic temperature, and *N*_*e*_ is the percentage of valence electrons excited to the conduction band.

Material	Phase	Type of the transition	*T*_*e*_, eV	*N*_*e*_, %
Al_2_O_3_	α, R-3c	Superionic	2.75	4.8
	Ia-3^a^	Superionic	2.75	5.7
Ga_2_O_3_	α, R-3c, PBE	Superionic	2.25	4.9
	α, R-3c, SCAN	Superionic	2.5	5.1
	β, C2/m	Melting	1.75	3.4
	δ, Ia-3	Superionic	2.25	5.3
In_2_O_3_	rh, R-3c	Superionic	2	5.3
	c, Ia-3	Superionic	2	5.4
In_2_S_3_	ε, R-3c	Melting	1.25	5.5

^a^To the best of our knowledge, Ia-3 phase of Al_2_O_3_ appears only in simulations^29^. It was studied here for the sake of comparison of different materials with the same space group symmetry.

For all materials becoming superionic during nonthermal transition (except for c-In_2_O_3_ discussed below) we observed the same profile of mean atomic displacements. During the first ~ 250–300 fs, both metal (Me) and O atoms are rapidly moving away from their ambient equilibrium positions. After that, displacements of Me atoms saturate at values ~ 0.6 Å, while O atoms keep moving demonstrating diffusive (liquid-like) behavior.

**Figure 1:**
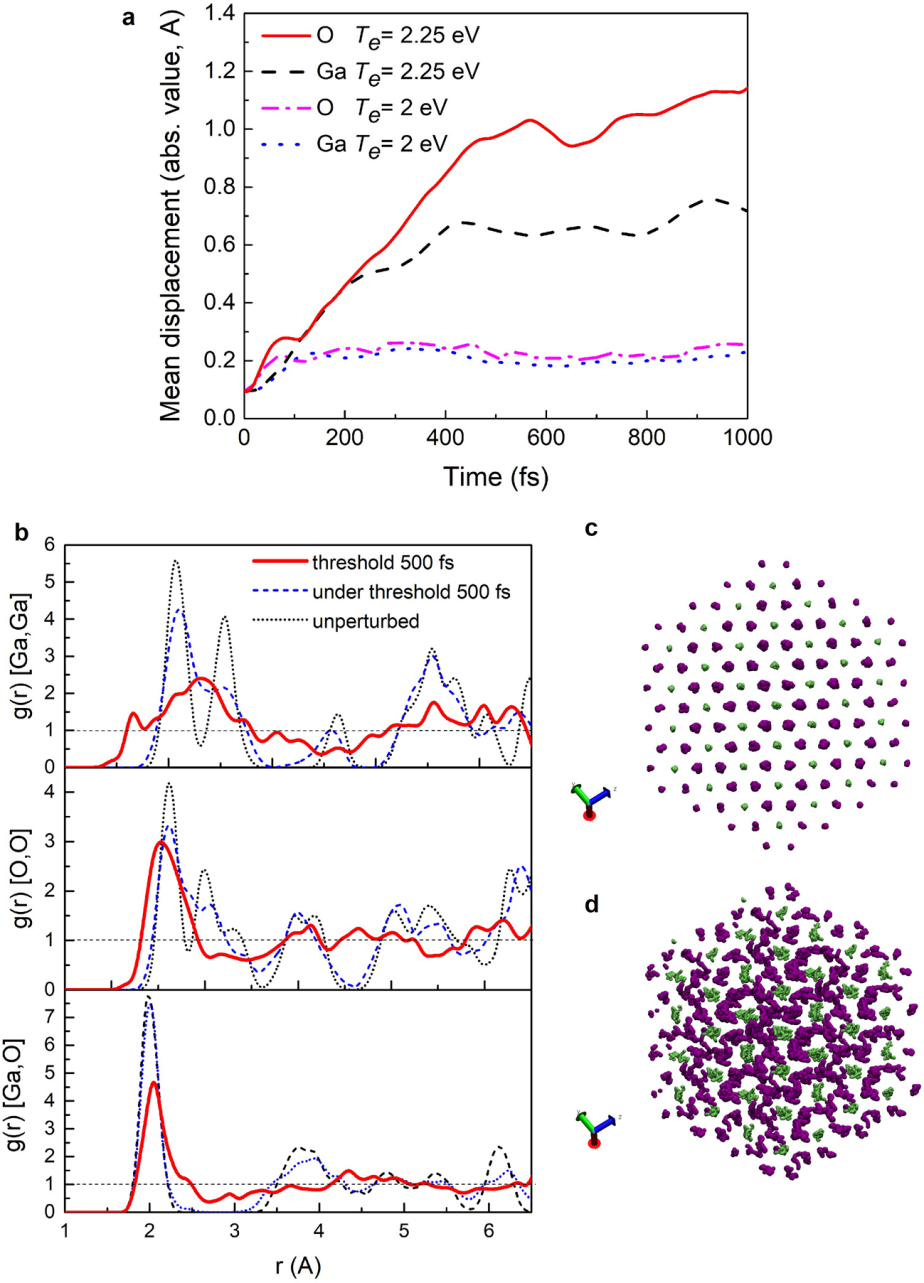
(**a**) Mean atomic displacements in Ia-3 phase of Ga_2_O_3_ at the electronic temperature (2 eV) below the superionic threshold and at the threshold electronic temperature (2.25 eV); (**b**) Radial pair distribution functions of unperturbed Ia-3 phase of Ga_2_O_3_ and those at the electronic temperature (2 eV) below the superionic threshold and at the threshold electronic temperature (2.25 eV); (**c**) atomic trajectories in one supercell of R-3c phase of Ga_2_O_3_ at the electronic temperature (2 eV) below the superionic threshold; (**d**) Atomic trajectories during 500 fs of simulation in one supercell of R-3c phase of Ga_2_O_3_ at the threshold electronic temperature (2.25 eV).

An example of the radial pair distribution functions (RPDF) and atomic trajectories in a supercell are presented in Fig. 1. One may see that O–O RPDF demonstrates typical liquid-like behavior at threshold temperature, while Ga–Ga RPDF resembles that of a damaged material—broad main peak and a number of smaller peaks with comparable magnitude. At the same time Ga–O RPDF demonstrates significant decrease of the main peak as well as vanishing of smaller peaks indicating weakening of correlations between Ga and O atoms.

In Fig. 1c,d one may see a difference in atomic trajectories (up to 500 fs) at the threshold temperature and below it for R-3c phase of Ga_2_O_3_. It is also clearly seen that at the threshold temperature Ga atoms (green) oscillate around their ambient equilibrium positions, while O atoms (purple) move more chaotically unbound from their former equilibrium positions. For clarity, these Figures represent 640 atoms supercells—translated original simulated supercells of 80 atoms. This and the choice of R-3c phase of Ga_2_O_3_ was done for the best visualization of a superionic state.

Additionally, we estimated ionic conductivities of materials becoming superionic via Nernst–Einstein relation^30^ and obtained values of some tens of mS/cm which is in a range of typical conductivities for superionic materials^31^.

In the Supplementary Information we present detailed information about each transition as well as a comparison of the mean displacements calculated with R2SCAN and PBE functional. R2SCAN predicts slightly higher electronic threshold temperature due to a larger band gap. However, the mean displacements calculated with PBE and R2SCAN are in a good agreement at the threshold temperatures. This confirms applicability of the used PBE functional for the purposes of this work.

We note that for Ia-3 phase of In_2_O_3_, nonthermal transition to the superionic state at *T*_*e*_ = 2 eV can be clearly identified only at times > 1 ps which is far longer than times at which the nonthermal transition channel can exist without noticeable interference from the thermal one; we thus expect that it may not be observable in experiments at the threshold temperature. However, increase of the electronic temperature to *T*_*e*_ = 2.25 eV results in much faster transition within < 500 fs, which should be observable.

Table 1 shows that the space group is not a main parameter driving the transition to a superionic state. In_2_S_3_ nonthermally melts, while other compounds with the same space group become superionic. Y_2_O_3_ (Ia-3 space group) in our previous study also did not demonstrate existence of a superionic state^4^.

A mere presence of oxygen atoms in a structure does not guarantee appearance of the superionic state, since C2/m phase of Ga_2_O_3_ exhibits melting, while all other studied materials with oxygen turn into superionic state.

Nevertheless, Table 1 confirms the idea that nearly all group III metal oxides can exhibit superionic behavior after ultrafast sufficient electronic excitation, although some irregularities occur in certain materials or their polymorphs. Thus, it seems that the origin of the ability to transform into a superionic material does not lie in the plane of simple properties such as atomic structure and chemical composition, and an analysis of electronic properties is required.

### Electronic properties

We compared calculated projected electronic density of states (PDOS) of each ambient material from Table 1 (all these PDOS can be found in Supplementary Information). Although all PDOS have some similarities and differences, there does not seem to be a definitive characteristic feature that differentiate C2/m phase of Ga_2_O_3_ and In_2_S_3_ from other materials and that could be interpreted as an unambiguous indicator of an ability or inability of a material to exhibit transition to the superionic state. This suggests that electronic energy levels structure itself does not determine a type of a transition. Instead, a complex interplay of various material properties affects formation of the superionic state.

**Figure 2:**
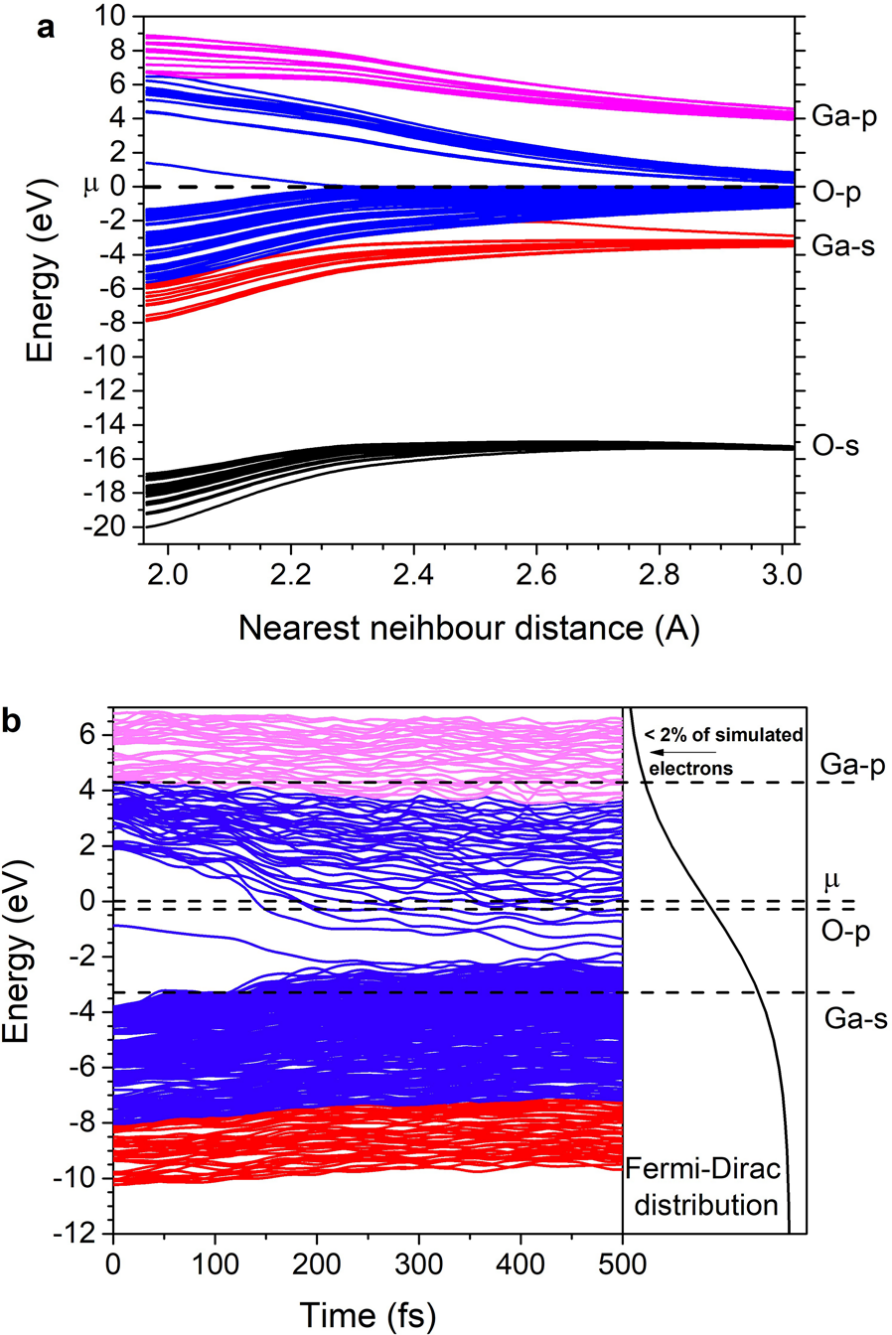
(**a**) Dependence of gamma-point energy levels of Ia-3 phase of Ga_2_O_3_ on interatomic distance; (**b**) Gamma-point energy levels dynamics during nonthermal transition into the superionic state. Here, μ is the chemical potential that is set to zero.

Figure 2a demonstrates a dependence of gamma-point energy levels in Ia-3 phase of Ga_2_O_3_ on the interatomic distance. It is clear that the upper energy levels of the valence band and the lower levels of the conduction band are formed from oxygen *p*-orbitals. This means that at elevated electronic temperatures triggering the transition into the superionic state, electrons are mainly excited from oxygen bonding energy levels that are shifted below those of isolated atom to oxygen antibonding (shifted above) levels while occupation numbers of metal bonding and antibonding levels remain almost untouched. Moreover, electronic temperature and atomic movement cause these levels to shift (Fig. 2b) making some of the *p*-levels of Ga (marked as “Ga-*p*”) to become bonding. By the end of the simulation (500 fs) only ~ 1.8% of simulated electrons are occupying “Ga-*p*” antibonding levels. All this indicates that the electronic excitation is affecting potentials in a way that may lead to oxygen melting while preserving metallic sublattice.

Further electronic temperature increase should involve more Ga energy levels both in the valence and conduction bands resulting in nonthermal melting of the material or at least significant changes in the Ga sublattice. This is exactly what happened with alumina in our previous study^12^.

Energy levels behavior from Fig. 2a is qualitatively the same for all materials and phases from Table 1. This means that C2/m phase of Ga_2_O_3_ as well as In_2_S_3_ should also have been transformed into a superionic state if the above reasoning is sufficient. Considering that these phases demonstrate melting instead of the superionic behavior, it means more than one material property affects the superionic state formation.

### Potential energy surfaces

Analyzing properties of materials from Table 1, one finds that ε-In_2_S_3_ is a very unstable material that is starting to turn into β-In_2_S_3_ already at 40 °C^32^. This means that atoms in ε-In_2_S_3_ are in a very shallow potential well. In such a case, almost any significant external perturbation would lead to destabilization of the material.

In contrast, C2/m is the most stable phase of Ga_2_O_3_, while Ia-3 phase is more “exotic” and less stable at ambient conditions (it turns into ε-Ga_2_O_3_ at > 500 °C)^33^. Nevertheless, C2/m phase nonthermally melts in contrast to Ia-3 phase exhibiting solid-superionic phase transition. This difference may arise because of asymmetry in Ga potential surface that may result in preferential direction for atomic movement. In combination with thermal oscillations and interatomic potential changes after laser irradiation, this may be a source of easier destabilization of Ga lattice and consequently may lead to melting instead of a transition into superionic state after the electronic temperature elevation.

**Figure 3:**
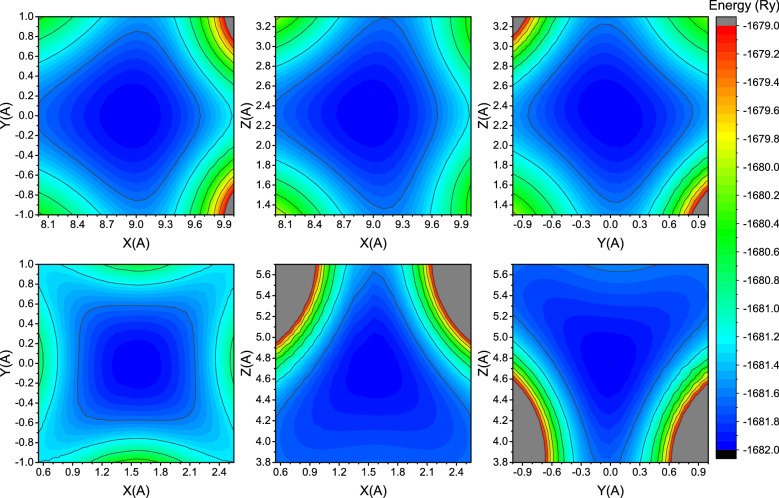
Potential energy surfaces of Ga atoms in Ia-3 phase (upper row) and C2/m phase (lower row) projected onto different planes of Ga_2_O_3_.

To illustrate this difference in Ga_2_O_3_ phases, we constructed potential energy surfaces of Ga atoms for Ia-3 and C2/m polymorphs (Fig. 3).

Figure 3 shows that indeed in C2/m phase, the potential energy surface of Ga is much more asymmetric and has highly distinctive preferential directions. This asymmetry also could be suspected from analysis of the macroscopic material properties: β-Ga_2_O_3_ has a significantly different thermal conductivity along different axes^34^.

**Figure 4:**
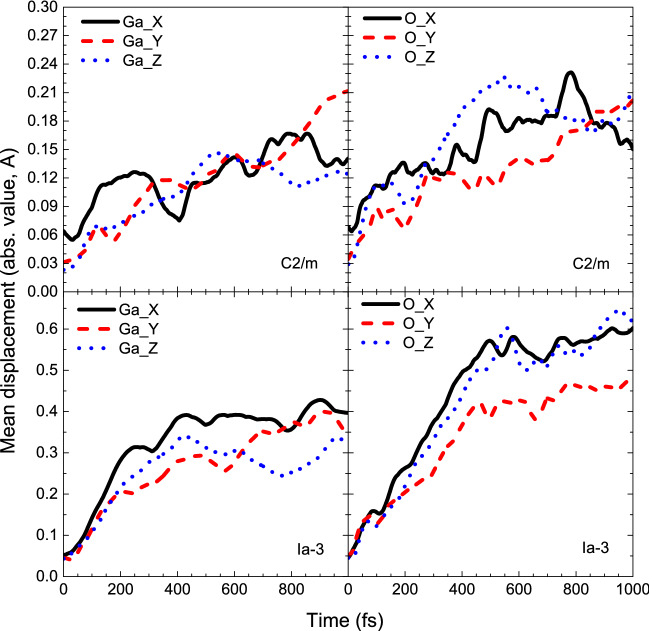
Mean atomic displacements along lattice vectors in C2/m phase (top) and Ia-3 phase of Ga_2_O_3_ (bottom) at threshold temperatures.

Figure 4 shows that Ga atoms in C2/m phase mainly move along *x* axis during the first ~ 200–250 fs with displacements twice as large as those along *y* and *z* axes. These displacements, however, are significantly smaller than displacements of metal atoms for any other material considered in this work or in our previous papers^4,12^. This also indicates that C2/m phase of Ga_2_O_3_ is an anomaly in the sense of nonthermal transitions.

## Discussion

**Figure 5:**
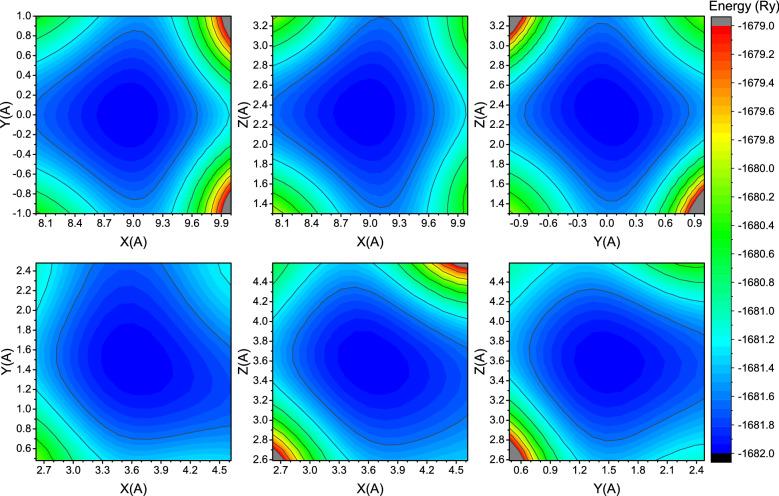
Potential energy surfaces of Ga (upper row) and O (lower row) atoms in Ia-3 phase of Ga_2_O_3_ at ambient conditions.

To elucidate a path of the nonthermal transition into a superionic state, we constructed potential energy surfaces of Ga and O atoms in Ia-3 phase at ambient conditions and at elevated electronic temperature at the initial moment of the transition (see Fig. 5).

As mentioned above, Ga atoms lie at a symmetric potential energy surface. At the same time, oxygen potential energy surface has clearly visible preferential directions along Y and X axes. This is confirmed by the mean displacements of oxygen presented in Fig. 4: within the first ~ 100 fs oxygen moves primarily along X and Y axes until potential energy surface profile will change and movement along X and Z axes will become dominant.

Also energy isosurfaces (constant energy surfaces) in all pictures are wider for O atoms. This means that the oxygen potential well is slightly shallower than the gallium one allowing for easier displacements of oxygen atoms.

**Figure 6:**
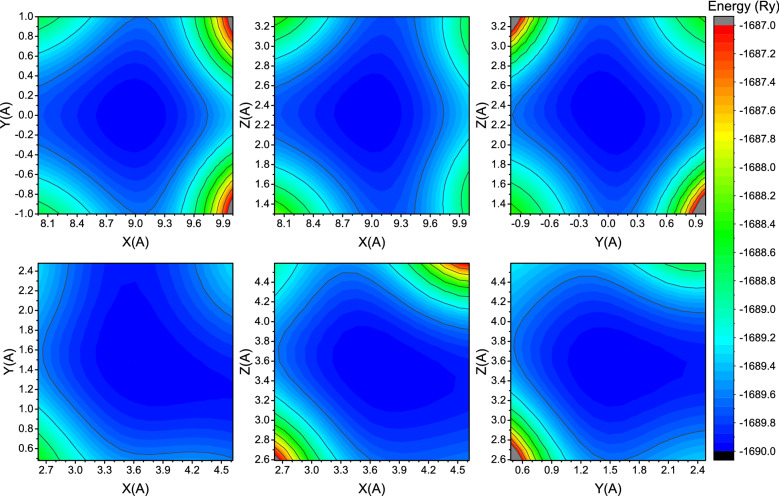
Potential energy surfaces of Ga (upper row) and O (lower row) atoms in Ia-3 phase of Ga_2_O_3_ at threshold electronic temperature at the initial time instant.

Increase of the electronic temperature to the values around the superionic threshold itself does not seem to change the potential energy surface qualitatively—it simply makes the potential well shallower (see Fig. 6). It is important that an oxygen potential energy surface seems to change more drastically: one can see that oxygen energy isosurfaces, which fit inside Fig. 5, went out of bounds in Fig. 6 while gallium energy isosurfaces changed only slightly. An asymmetry of the potential energy surface may help a material to form superionic channels for oxygen under extreme electronic excitation.

Combining obtained information, we can deduce the following path of nonthermal transitions into a superionic state in the investigated compounds. The transition starts from a fast displacement of O atoms because of shallow and highly asymmetric potential well^35^. Moving oxygen atoms change the potential energy surface profile of metal atoms “dragging” them away from the equilibrium positions of an ambient crystal. This “dragging” continues until metal atoms find positions not hindering further oxygen atoms flow. After that, although the mean displacement of metal atoms remains almost constant, they may strongly oscillate around new positions as it is seen from mean displacements along the lattice vectors (XYZ axes).

For the observed materials exhibiting transition into a superionic state, the mean displacement saturation level at the threshold electronic temperature for metal atoms is usually around ~ 0.6 Å while oxygen atoms are displaced to larger distances > 0.8 Å at 500 fs after the electronic temperature increase. This saturation of the metallic atoms displacements may result from energy levels changes during the transition: when a sufficient amount of metal levels turns its behavior from antibonding to bonding, metal atoms slow down settling in a new sublattice.

We presume that non-oxide III–VI group materials should be capable of transforming into a superionic state. However, the majority of these compounds have defective structures^36^ or even contradictory information exists about their crystal structure^37^. This makes them challenging for first-principles calculations^38^ and requires a separate dedicated study.

## Conclusion

We established threshold electronic temperatures triggering nonthermal phase transitions and their types (solid–liquid or solid-superionic) in a number of group III-metal oxides in the warm dense matter state. We demonstrated that the majority of considered materials exhibit nonthermal transitions into a superionic phase where oxygen exhibits a liquid-like behavior in contrast to the metallic sublattice remaining in a solid state.

We analyzed electronic structures of these materials and concluded that a unique combination of bonding and antibonding states may be responsible for such a behavior. A transition into a superionic state occurs when an asymmetry of oxygen potential energy surface induces liquid-like flow of oxygen atoms.

## Supplementary Information

Supplementary Information.
