# Supplementary material for: Superionic states formation in group III oxides irradiated with ultrafast lasers
Source: Sci Rep. 2022 Apr 5;12:5659. doi: 10.1038/s41598-022-09681-0 (PMC8983778; doi:10.1038/s41598-022-09681-0)
Supplement: Supplementary file 1 — Supplementary Information. [file 41598_2022_9681_MOESM1_ESM.pdf]

Supplementary materials for

"Superionic states formation in group III oxides irradiated with ultrafast lasers"

R.A. Voronkov[1*], N. Medvedev[2,3], A.E. Volkov[1]

[1]*P. N. Lebedev Physical Institute of the Russian Academy of Sciences, Leninskij pr., 53,119991 Moscow, Russia;*

[2]*Institute of Physics, Czech Academy of Sciences, Na Slovance 2, 182 21 Prague 8, Czech Republic;*

[3]*Institute of Plasma Physics, Czech Academy of Sciences, Za Slovankou 3, 182 00 Prague 8, Czech Republic;*

I. Information about nonthermal transitions

In this file, detailed information about nonthermal transitions from the main text is presented. In order to justify thresholds and transition type claimed in the manuscript, for each material mean atomic displacements and calculated X-ray diffraction (XRD) patterns are shown in Section I for the electronic temperatures below the transition threshold as well as those for the threshold. For R-3c phase of Ga_2O_3 mean displacements were also calculated with R2SCAN functional to confirm capability of PBE functional to reproduce correct qualitative behavior. XRD patterns of the simulated supercell are calculated via VESTA software. Mean displacements along lattice vectors at the threshold temperature are also presented to provide additional details about atomic kinetics during transitions. Projected electronic densities of states for each material are shown in Section II in order to demonstrate that PDOS do not contain distinctive features allowing to predict nonthermal transition behavior.

1. Al_2O_3 Ia-3

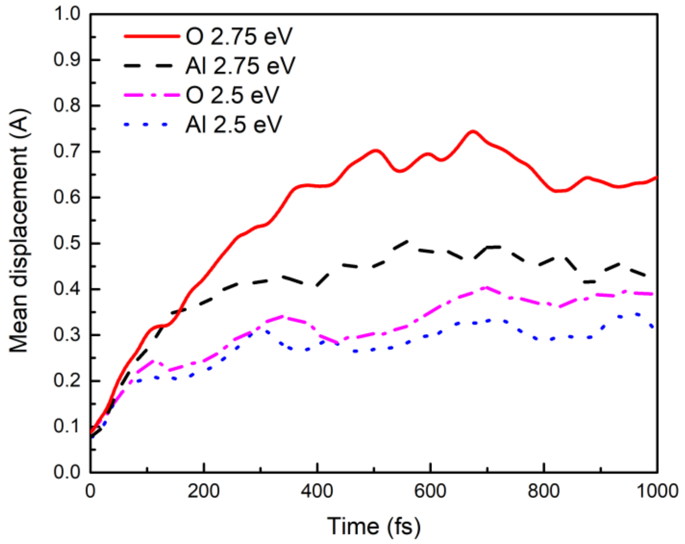

Figure 1.1.1. Mean atomic displacements at the electronic temperature (2.5 eV) below the threshold and at the threshold electronic temperature (2.75 eV). One can see saturation of Al displacements at T_e = 2.75 eV around 0.4 Å, while O atoms demonstrate liquid-like behavior.

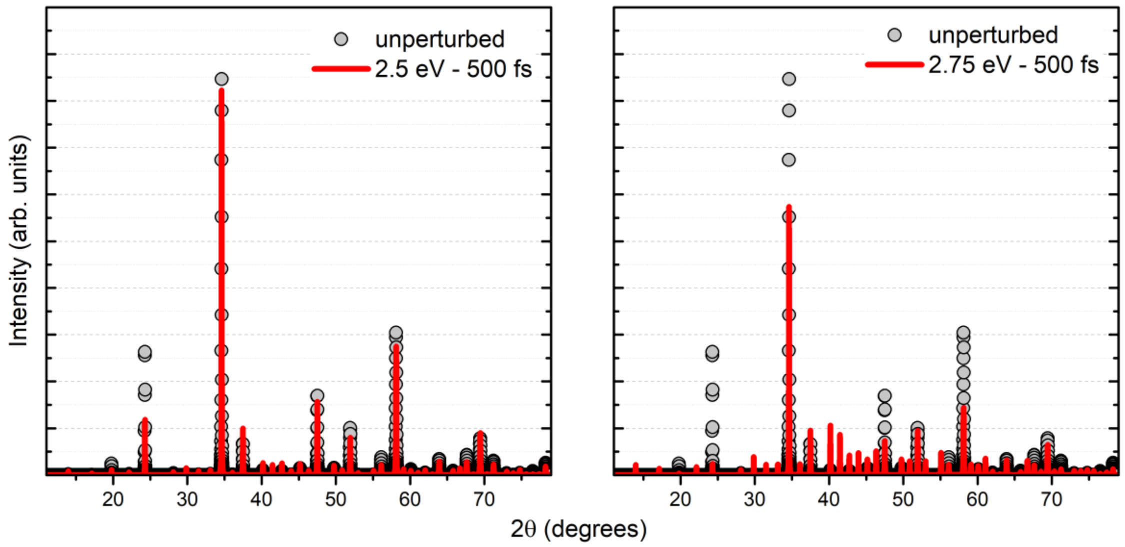

Figure 1.1.2. XRD patterns (λ=1.5406 Å) of the simulated supercell at T_e = 2.5 eV (below threshold) and T_e = 2.75 eV (threshold) at the initial and final time instants.

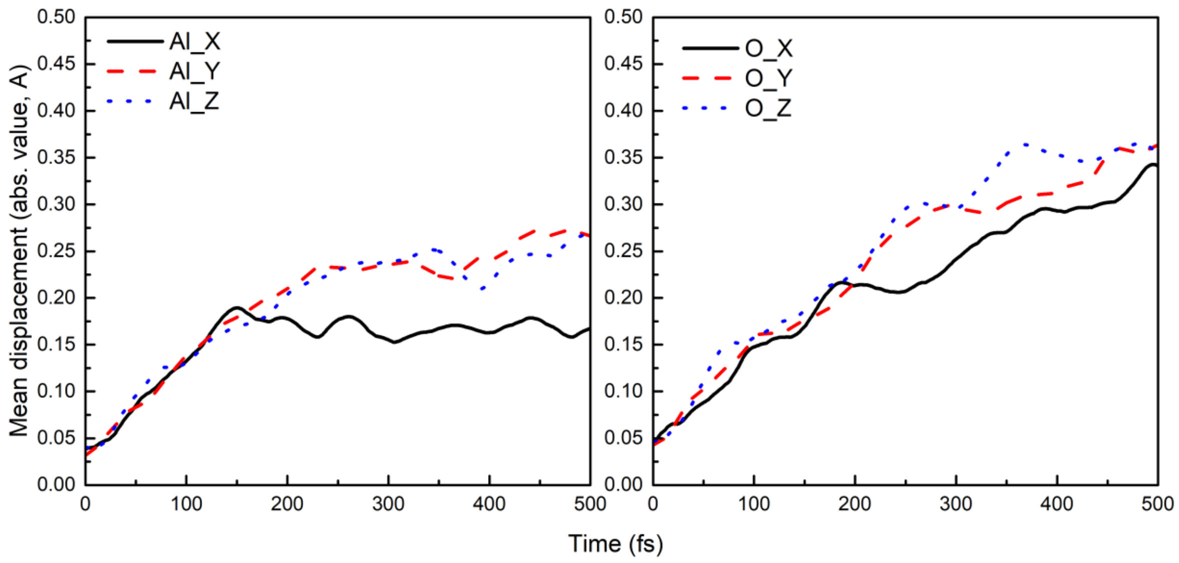

Figure 1.1.3. Absolute values of mean atomic displacements along lattice vectors at the threshold temperature T_e = 2.75 eV.

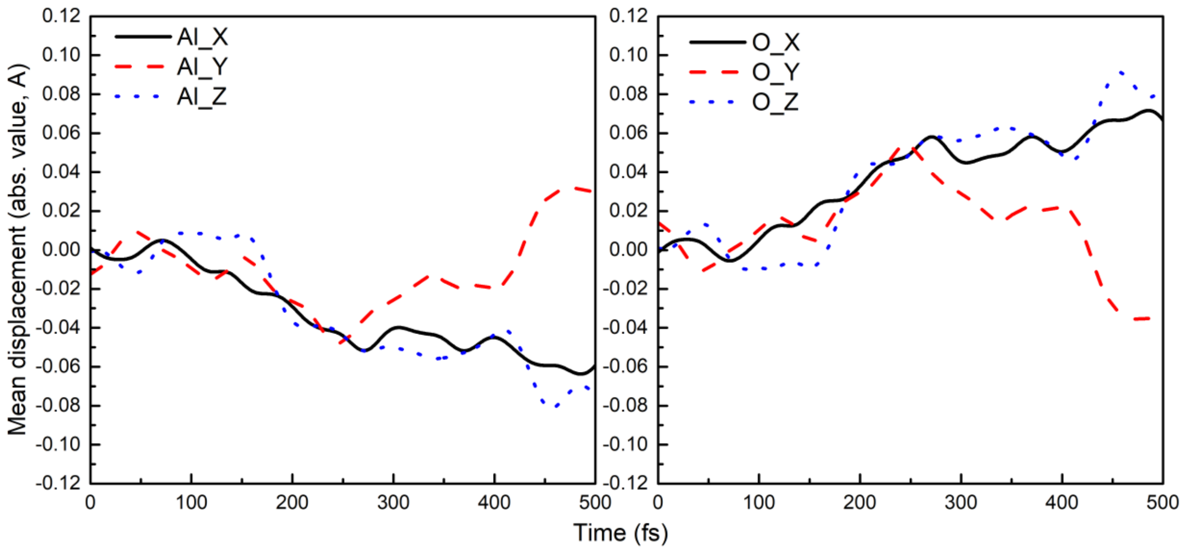

Figure 1.1.4. Mean atomic displacements along lattice vectors at the threshold temperature T_e = 2.75 eV. Negative values correspond to the movement against lattice vector direction.

2. Ga_2O_3 R-3c

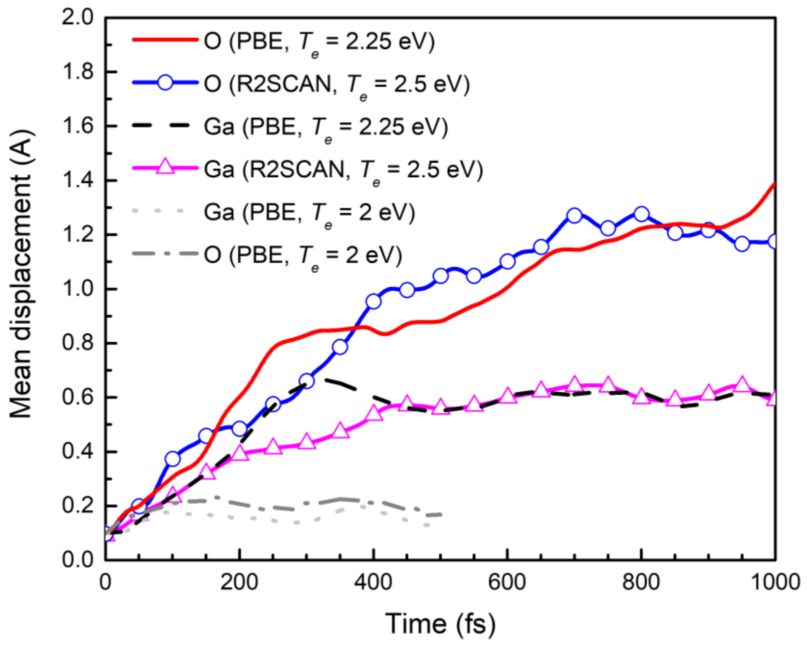

Figure 1.2.1. Mean atomic displacements at the electronic temperature (2 eV) below the threshold with PBE functional, at the threshold electronic temperature (2.25 eV) with PBE functional and at the threshold temperature (2.5 eV) with R2SCAN functional. One can see good qualitative agreement for PBE and R2SCAN functionals: saturation of Ga displacements at threshold temperatures around 0.6 Å, while O atoms demonstrate liquid-like behavior.

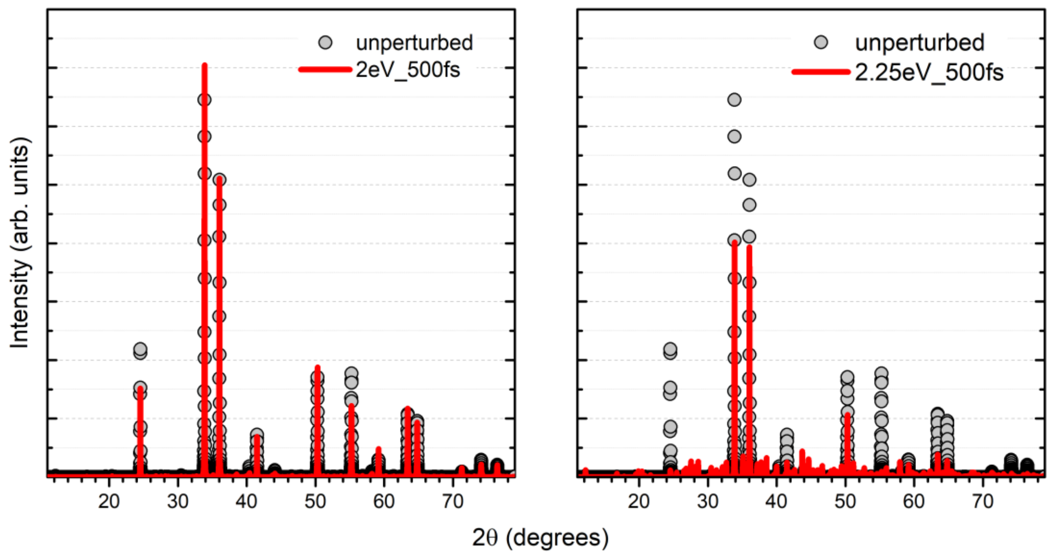

Figure 1.2.2. XRD patterns (λ=1.5406 Å) of the simulated supercell at T_e = 2 eV (below threshold) and T_e = 2.25 eV (threshold) at the initial and final time instants.

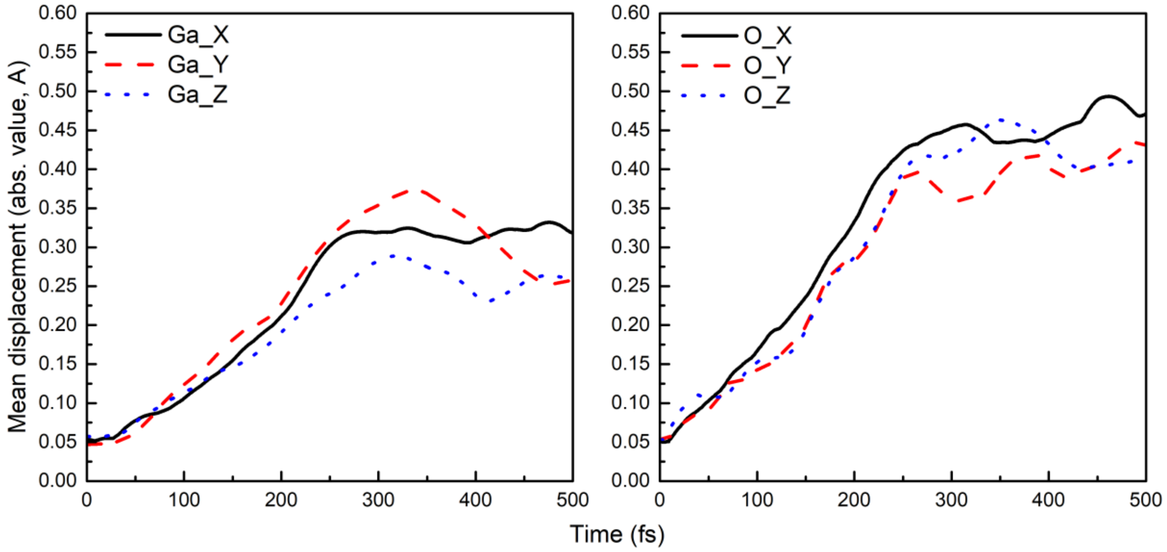

Figure 1.2.3. Absolute values of mean atomic displacements along lattice vectors at the threshold temperature T_e = 2.25 eV.

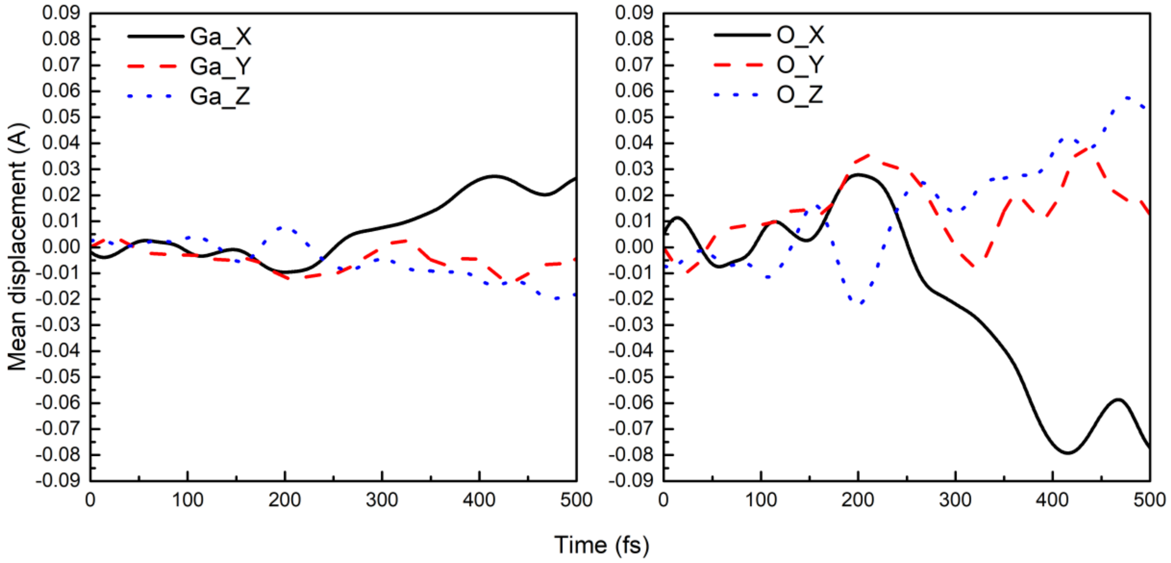

Figure 1.2.4. Mean atomic displacements along lattice vectors at the threshold temperature T_e = 2.25 eV. Negative values correspond to the movement against lattice vector direction.

3. Ga_2O_3 Ia-3

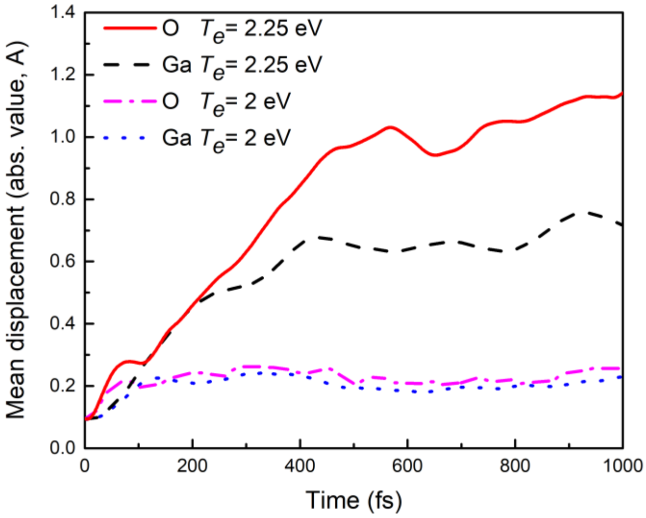

Figure 1.3.1. Mean atomic displacements at the electronic temperature (2 eV) below the threshold and at the threshold electronic temperature (2.25 eV). One can see saturation of Ga displacements at T_e = 2.25 eV around 0.6 Å, while O atoms demonstrate liquid-like behavior.

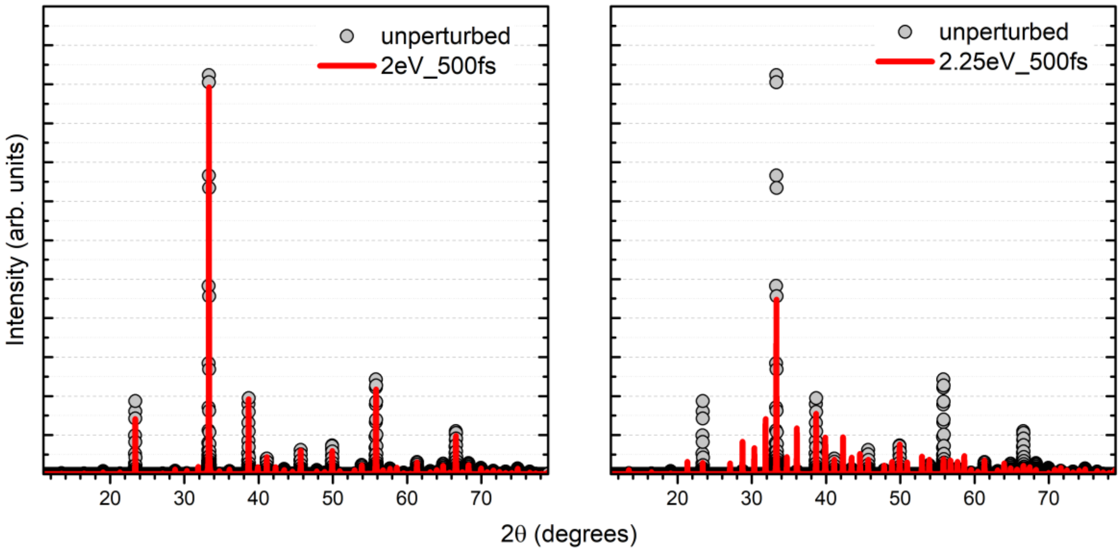

Figure 1.3.2. XRD patterns (λ=1.5406 Å) of the simulated supercell at T_e = 2 eV (below threshold) and T_e = 2.25 eV (threshold) at the initial and final time instants.

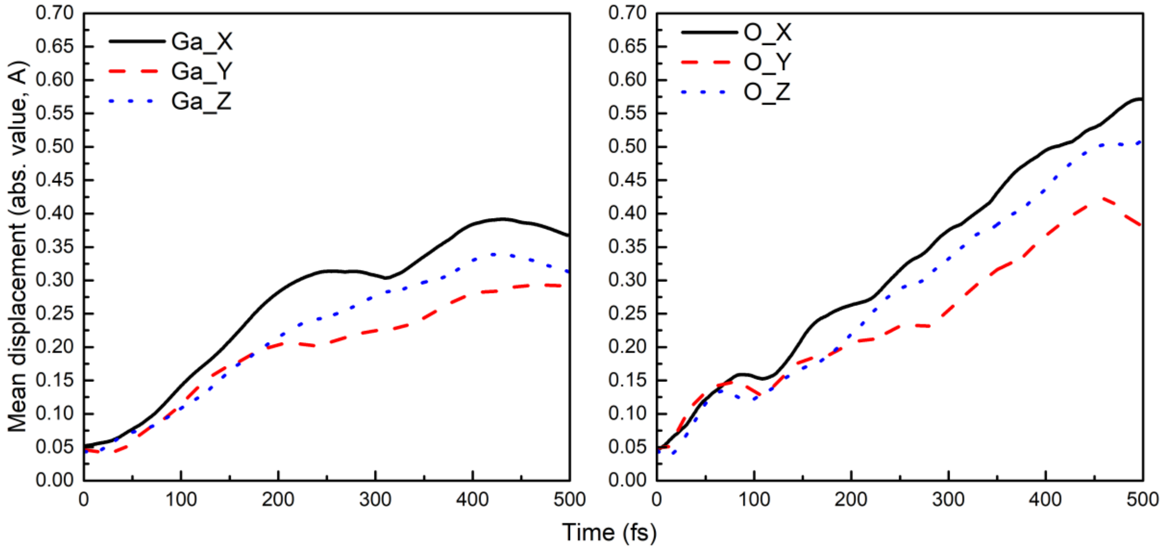

Figure 1.3.3. Absolute values of mean atomic displacements along lattice vectors at the threshold temperature T_e = 2.25 eV.

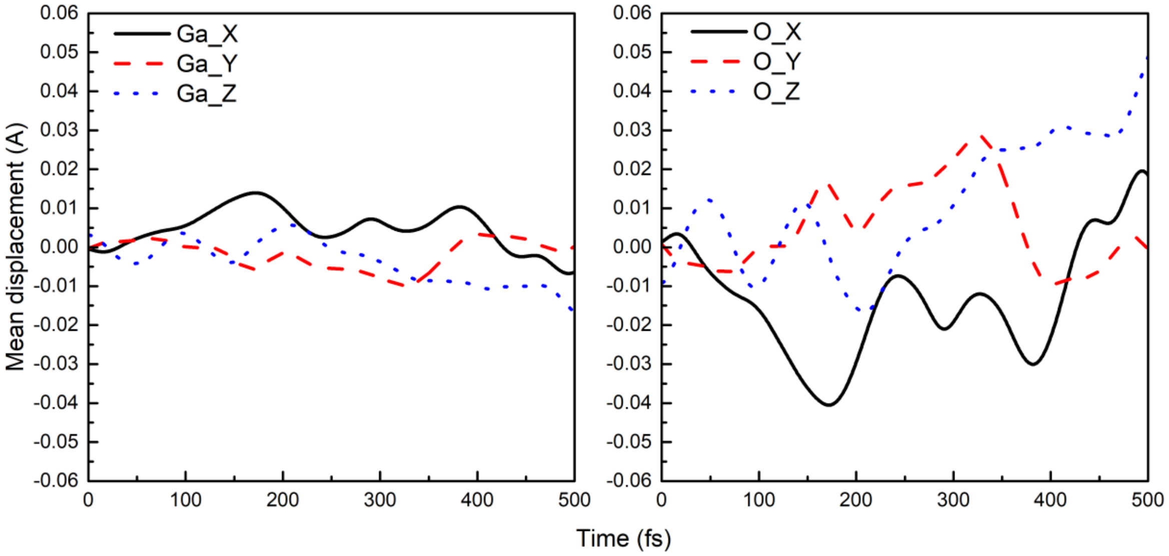

Figure 1.3.4. Mean atomic displacements along lattice vectors at the threshold temperature T_e = 2.25 eV. Negative values correspond to the movement against lattice vector direction.

4. Ga_2O_3 C2/m

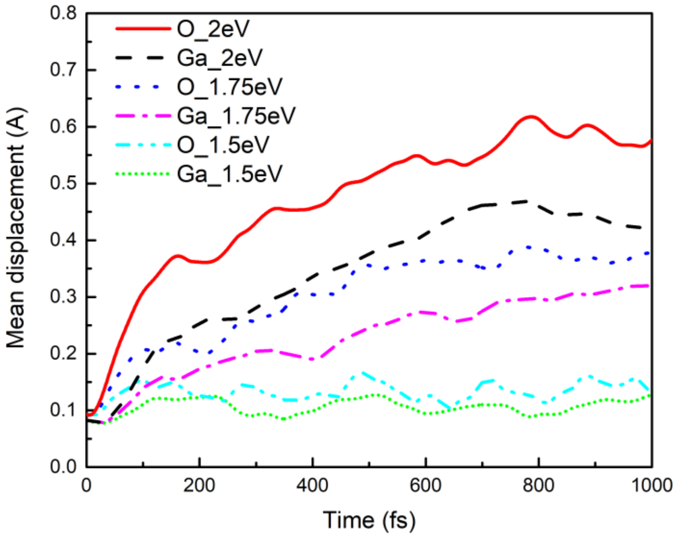

Figure 1.4.1. Mean atomic displacements at the electronic temperature (1.5 eV) below the threshold, at the threshold electronic temperature (1.75 eV) and the electronic temperature above threshold (2 eV). One can see an absence of displacements saturations indicating nonthermal melting.

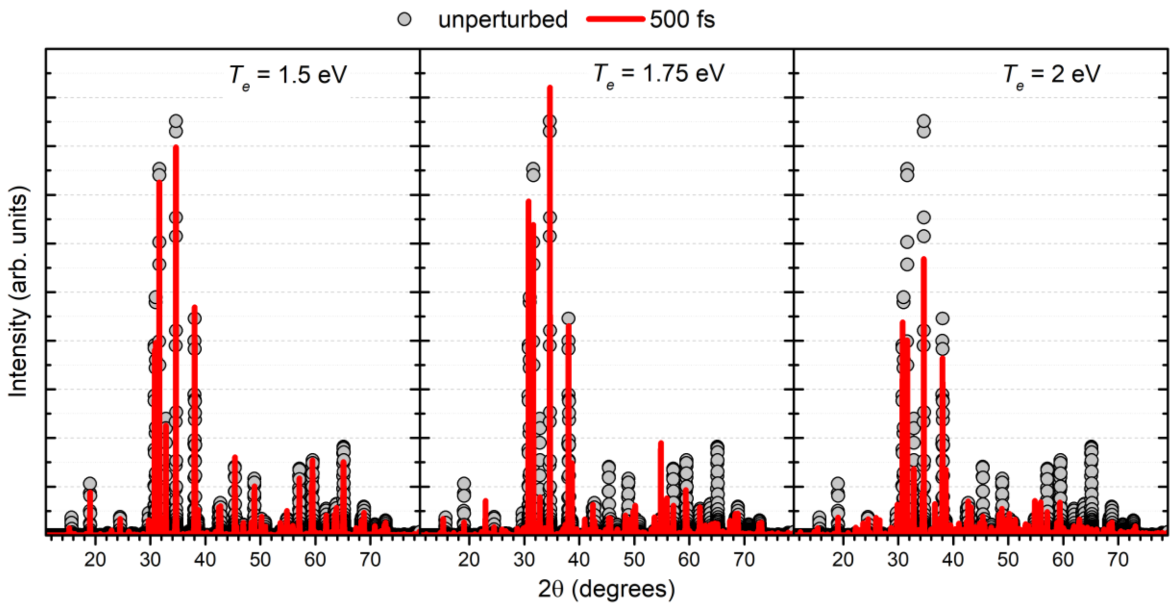

Figure 1.4.2. XRD patterns (λ=1.5406 Å) of the simulated supercell at the electronic temperature (1.5 eV) below the threshold, at the threshold electronic temperature (1.75 eV) and the electronic temperature above threshold (2 eV) at the initial and final time instants.

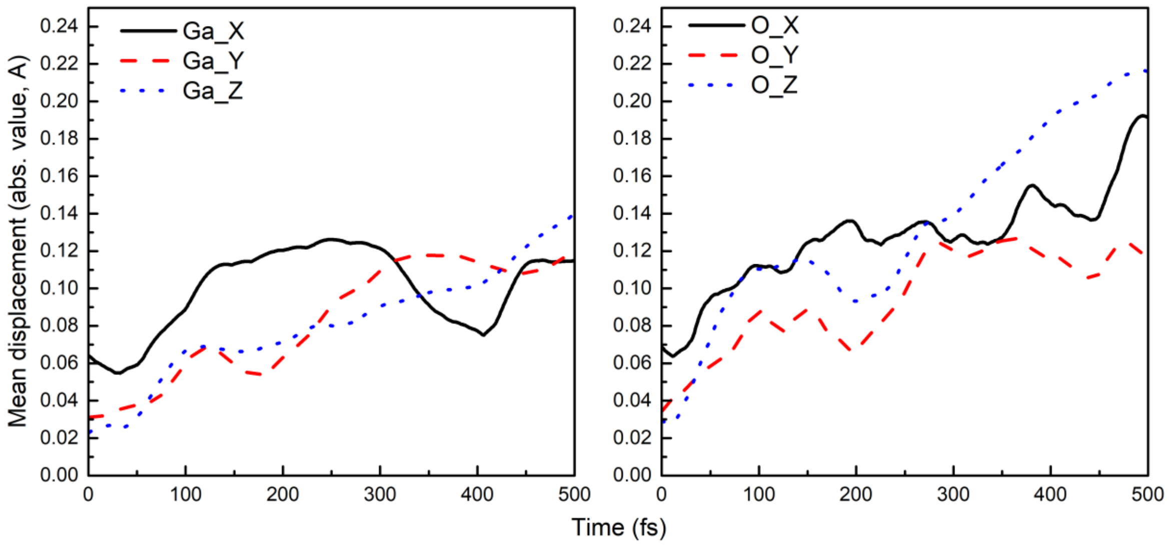

Figure 1.4.3. Absolute values of mean atomic displacements along lattice vectors at the threshold temperature T_e = 1.75 eV.

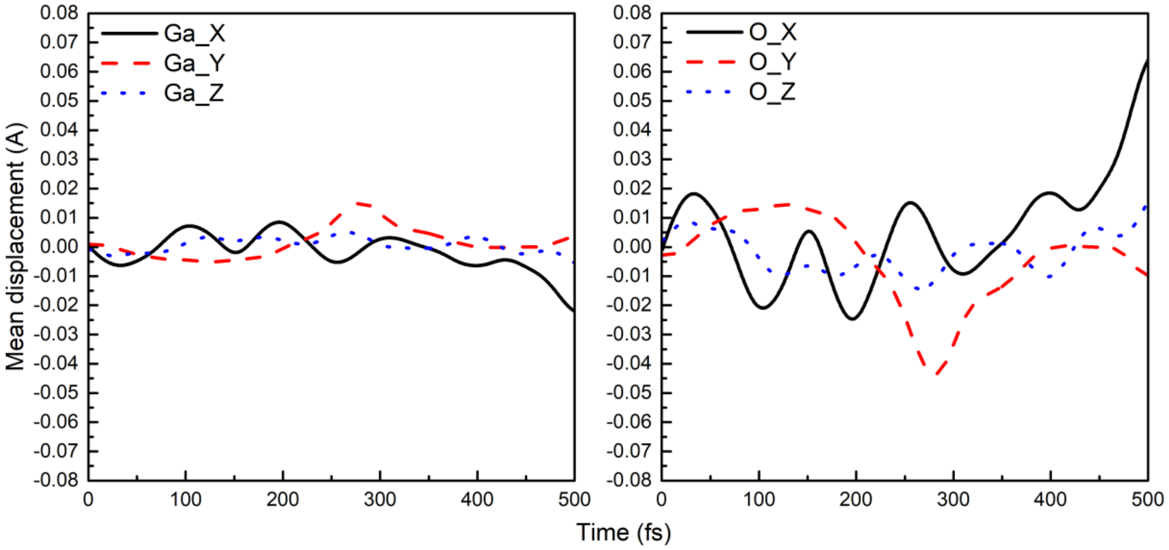

Figure 1.4.4. Mean atomic displacements along lattice vectors at the threshold temperature T_e = 1.75 eV. Negative values correspond to the movement against lattice vector direction.

5. In_2O_3 R-3c

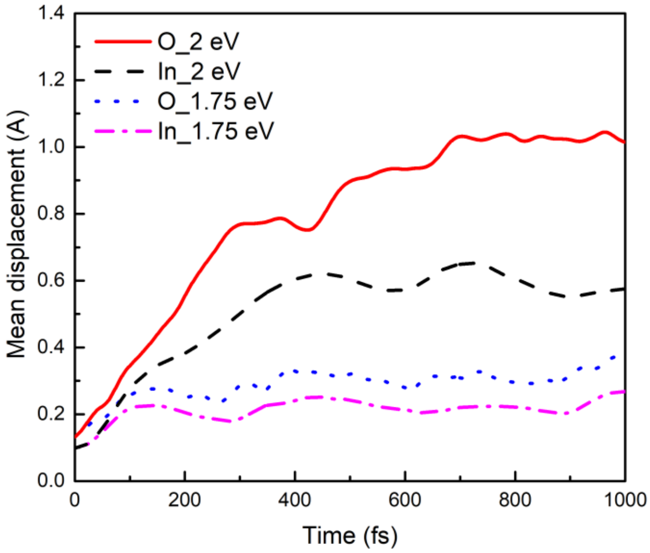

Figure 1.5.1. Mean atomic displacements at the electronic temperature (1.75 eV) below the threshold and at the threshold electronic temperature (2 eV). One can see saturation of In displacements at T_e = 2 eV around 0.6 Å, while O atoms demonstrate liquid-like behavior.

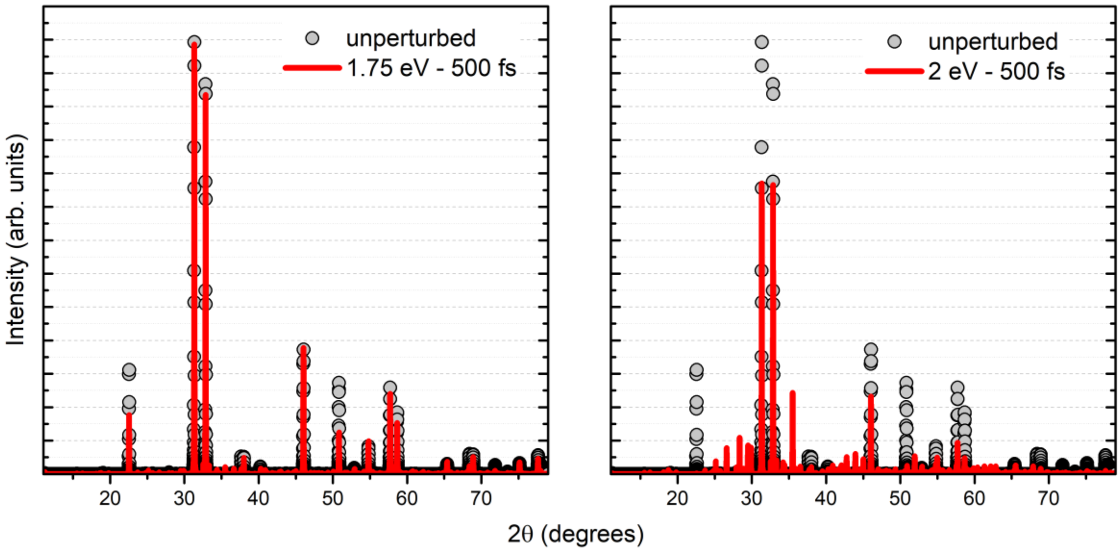

Figure 1.5.2. XRD patterns (λ=1.5406 Å) of the simulated supercell at T_e = 1.75 eV (below threshold) and T_e = 2 eV (threshold) at the initial and final time instants.

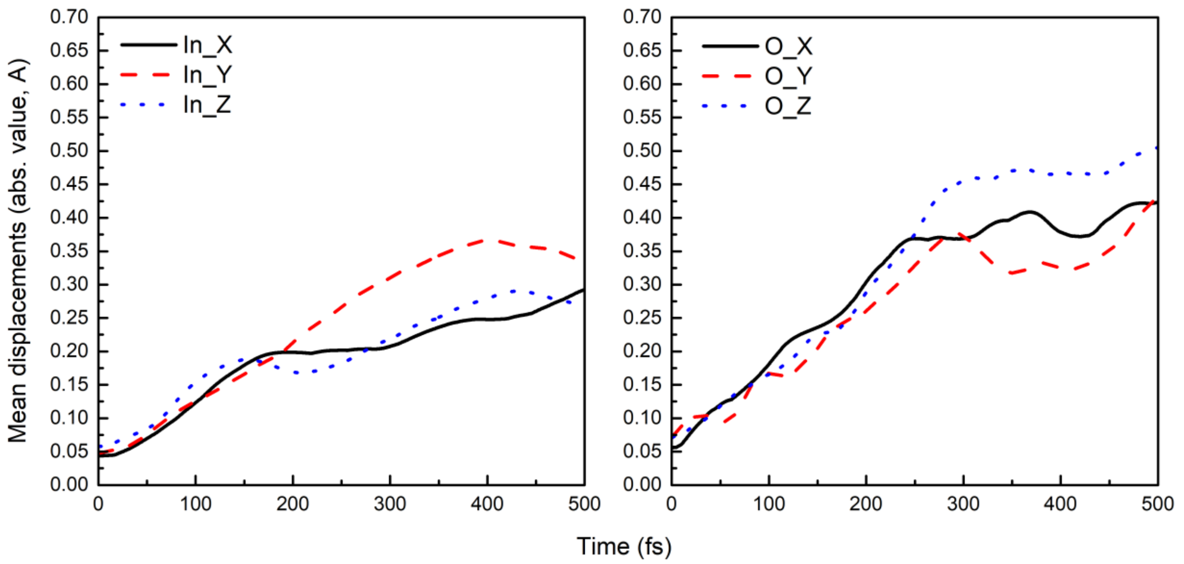

Figure 1.5.3. Absolute values of mean atomic displacements along lattice vectors at the threshold temperature T_e = 2 eV.

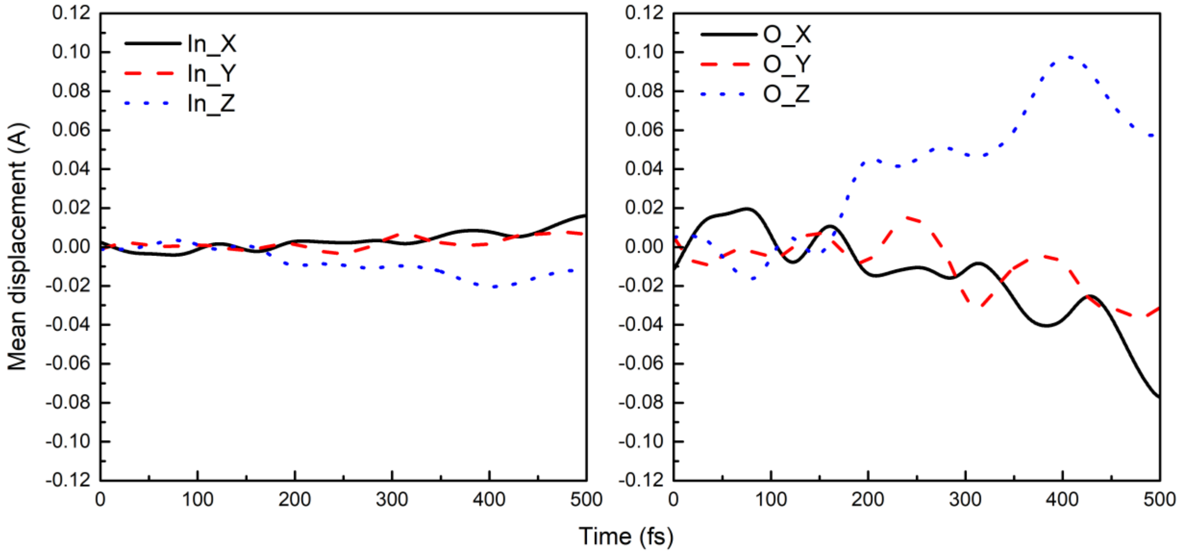

Figure 1.5.4. Mean atomic displacements along lattice vectors at the threshold temperature T_e = 2 eV. Negative values correspond to the movement against lattice vector direction.

6. In_2O_3 Ia-3

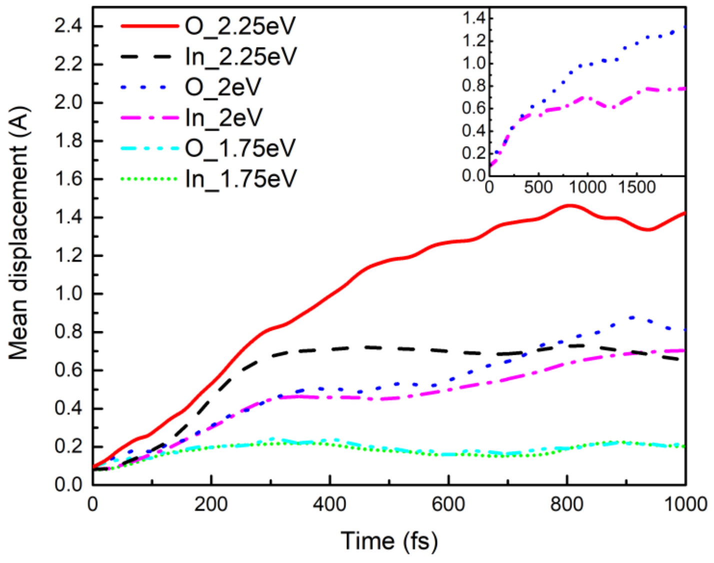

Figure 1.6.1. Mean atomic displacements at the electronic temperature (1.75 eV) below the threshold, at the threshold electronic temperature (2 eV) and the electronic temperature above the threshold one (2.25 eV). One can see saturation of In displacements at T_e = 2 eV and T_e = 2.25 eV around 0.6-0.7 Å, while O atoms demonstrate liquid-like behavior.

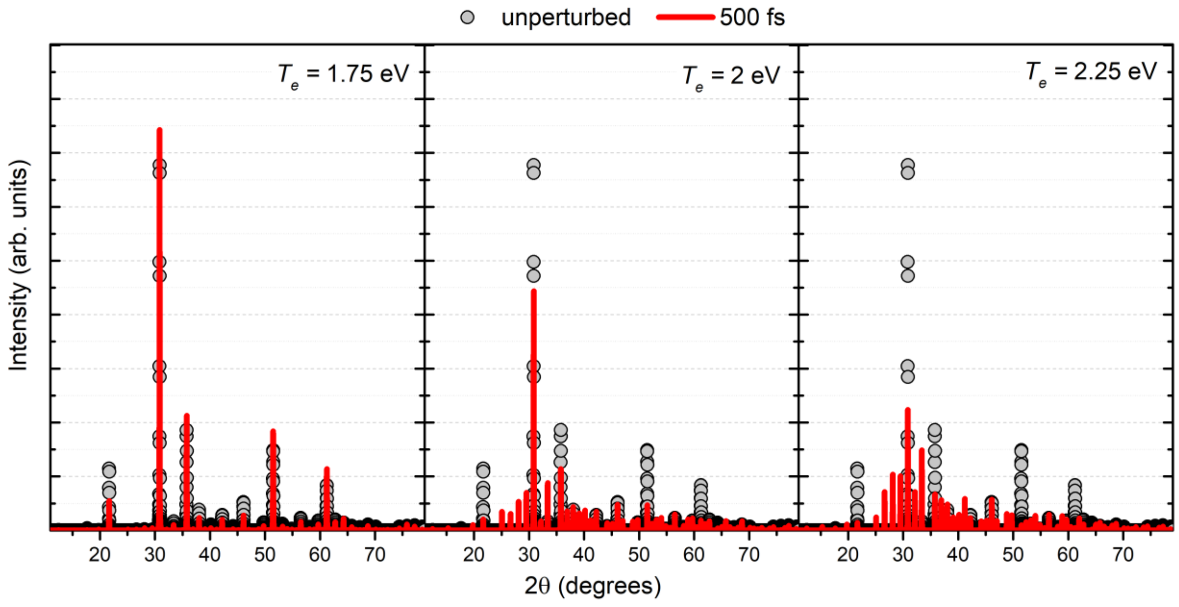

Figure 1.6.2. XRD patterns (λ=1.5406 Å) of the simulated supercell at the electronic temperature (1.75 eV) below the threshold, at the threshold electronic temperature (2 eV) and the electronic temperature above threshold (2.25 eV) at the initial and final time instants.

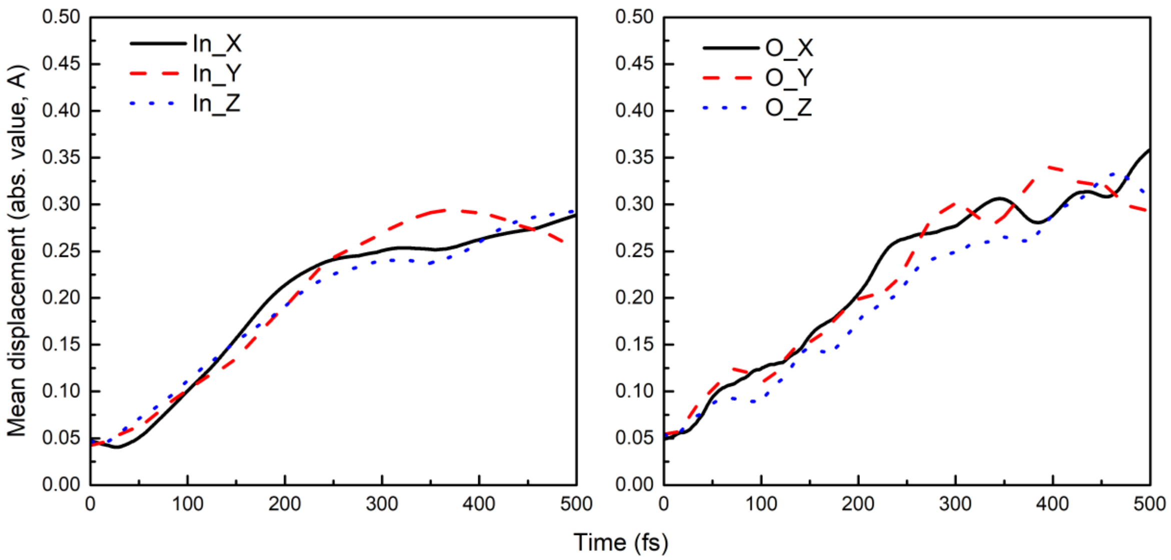

Figure 1.6.3. Absolute values of mean atomic displacements along lattice vectors at the threshold temperature $T_e = 2$ eV.

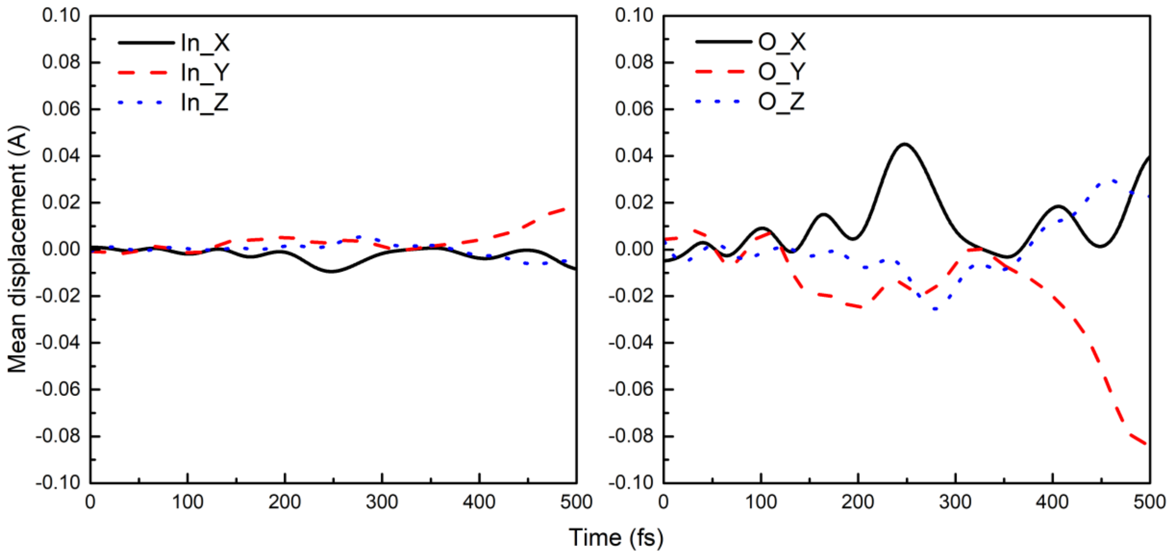

Figure 1.6.4. Mean atomic displacements along lattice vectors at the threshold temperature. Negative values correspond to the movement against lattice vector direction.

7. In_2S_3 R-3c

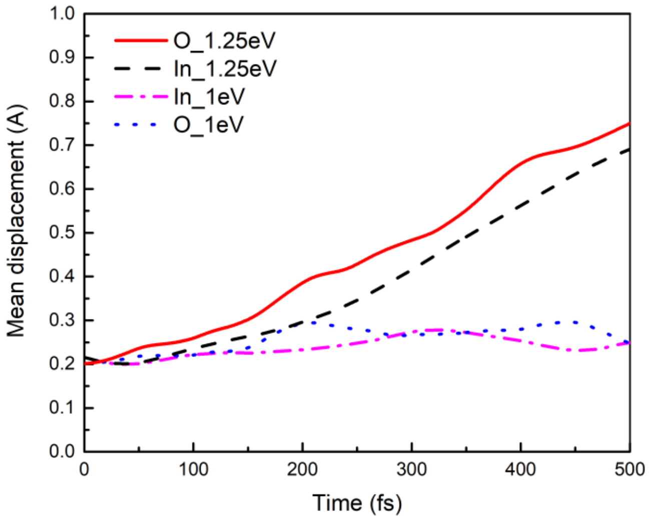

Figure 1.7.1. Mean atomic displacements at the electronic temperature (1 eV) below the threshold and at the threshold electronic temperature (1.25 eV). One can see an absence of displacements saturations indicating nonthermal melting.

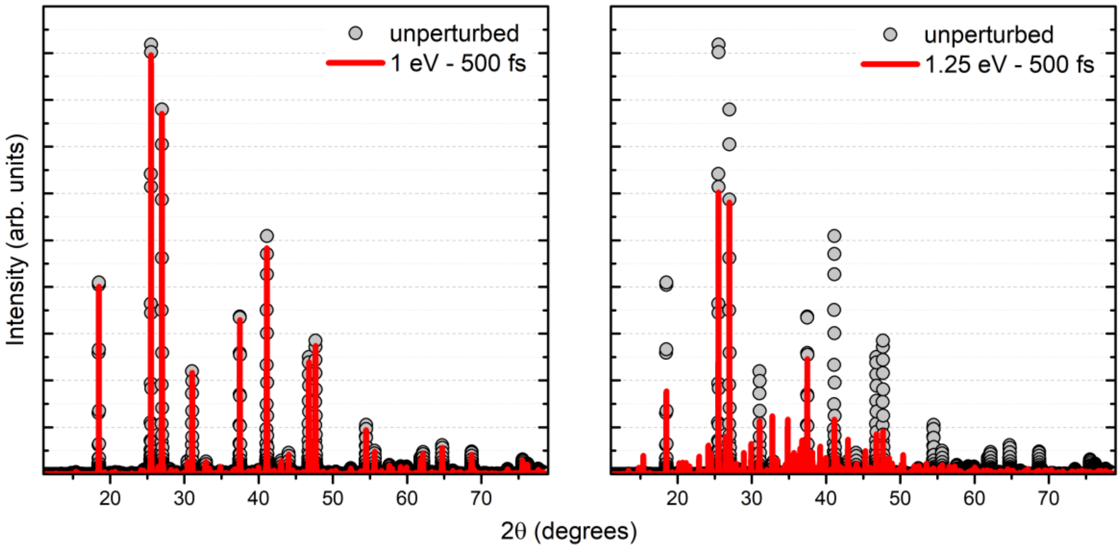

Figure 1.7.2. XRD patterns (λ=1.5406 Å) of the simulated supercell at T_e = 1 eV (below threshold) and T_e = 1.25 eV (threshold) at the initial and final time instants.

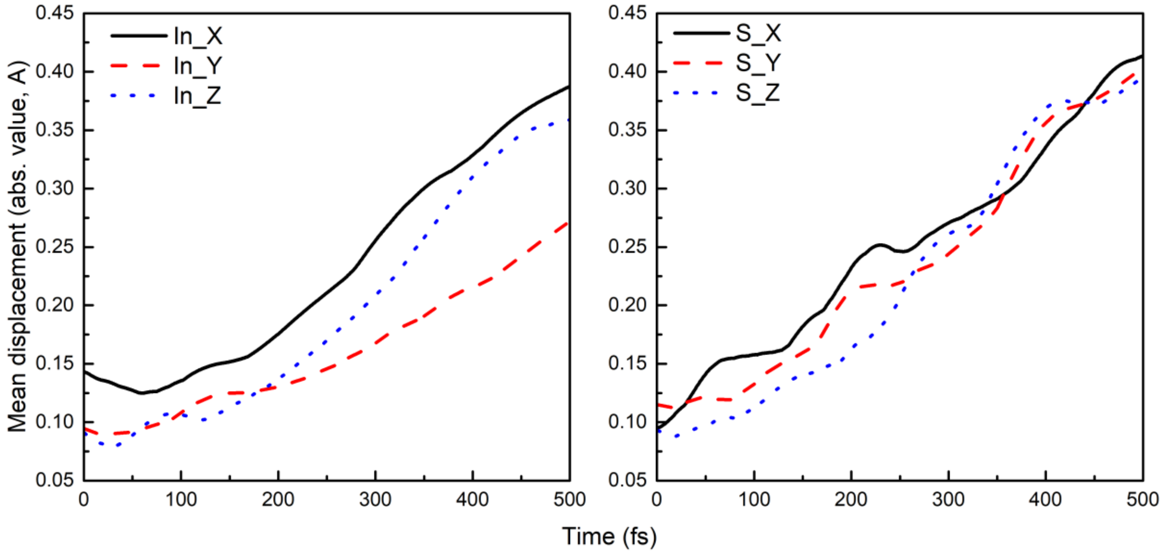

Figure 1.7.3. Absolute values of mean atomic displacements along lattice vectors at the threshold temperature T_e = 1.25 eV.

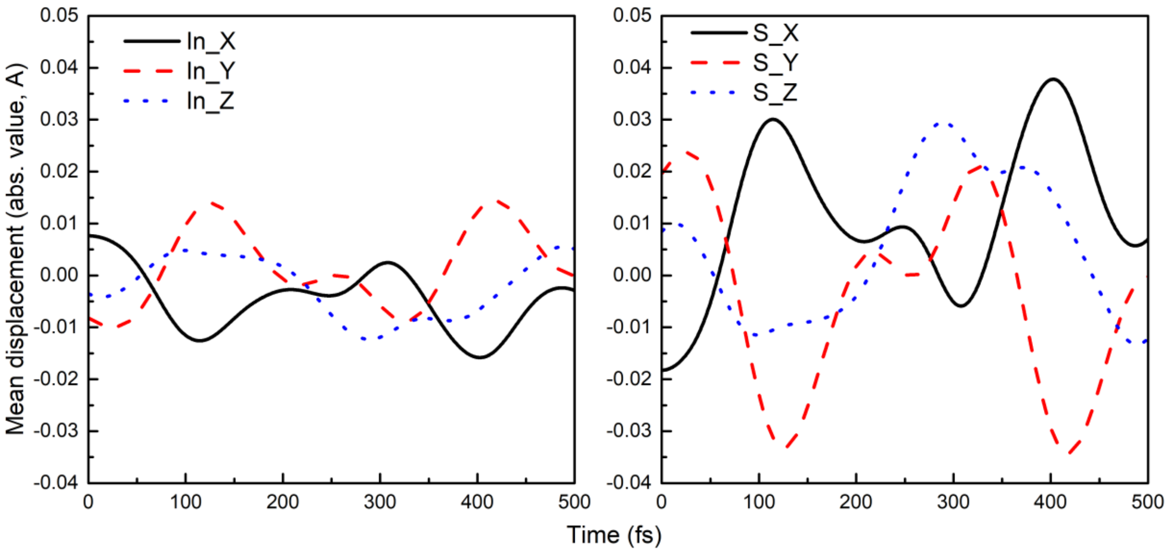

Figure 1.7.4. Mean atomic displacements along lattice vectors at the threshold temperature T_e = 1.25 eV. Negative values correspond to the movement against lattice vector direction.

II. Projected electronic density of states

1. Total contribution of O and Me atoms to electronic DOS

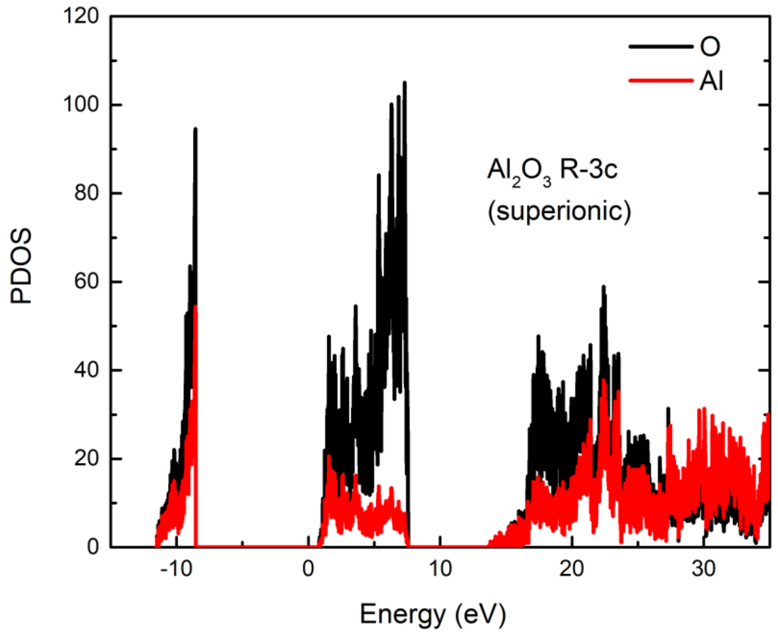

Figure 2.1.1. Total contribution of O and Al atoms to electronic DOS of R-3c phase of Al_2O_3.

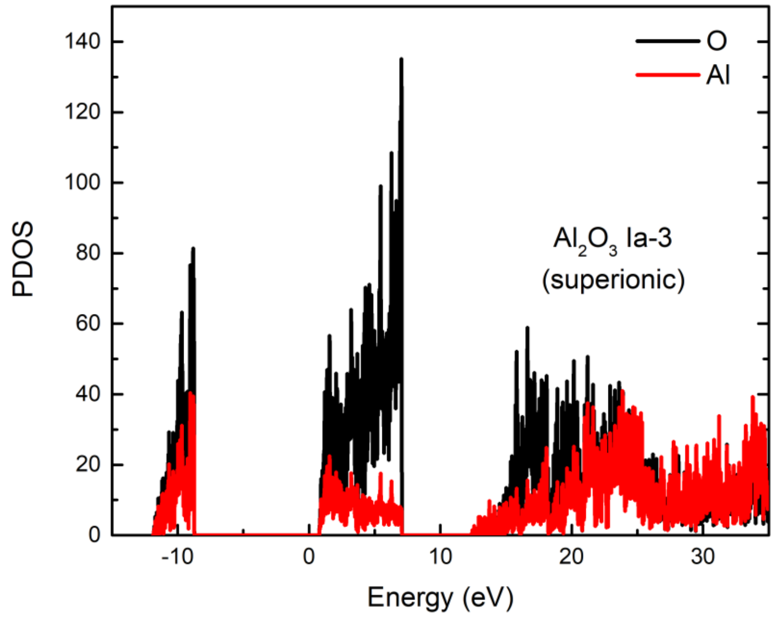

Figure 2.1.2. Total contribution of O and Al atoms to electronic DOS of Ia-3 phase of Al_2O_3.

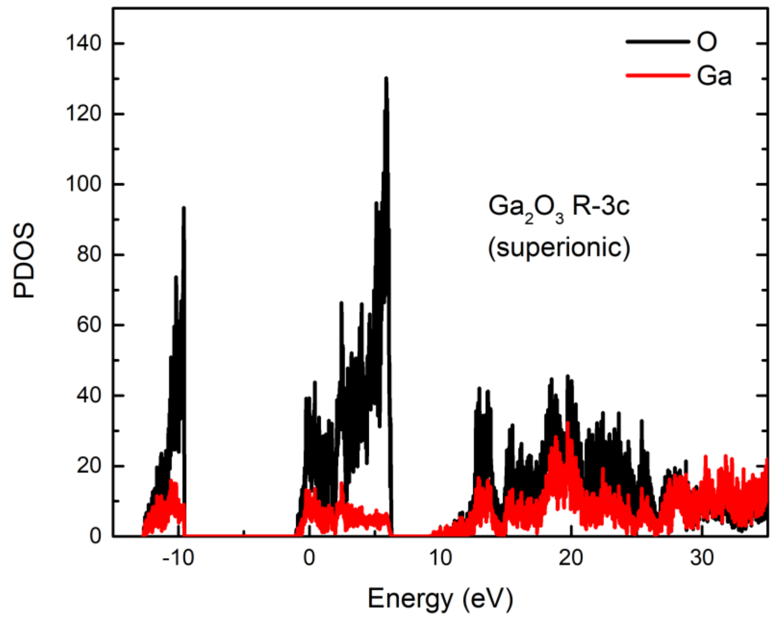

Figure 2.1.3. Total contribution of O and Ga atoms to electronic DOS of R-3c phase of Ga_2O_3.

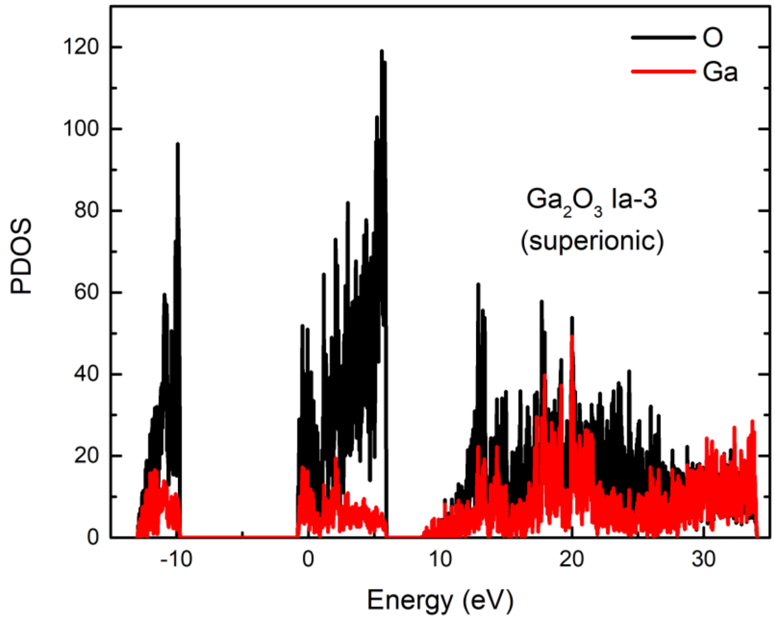

Figure 2.1.4. Total contribution of O and Ga atoms to electronic DOS of Ia-3 phase of Ga_2O_3.

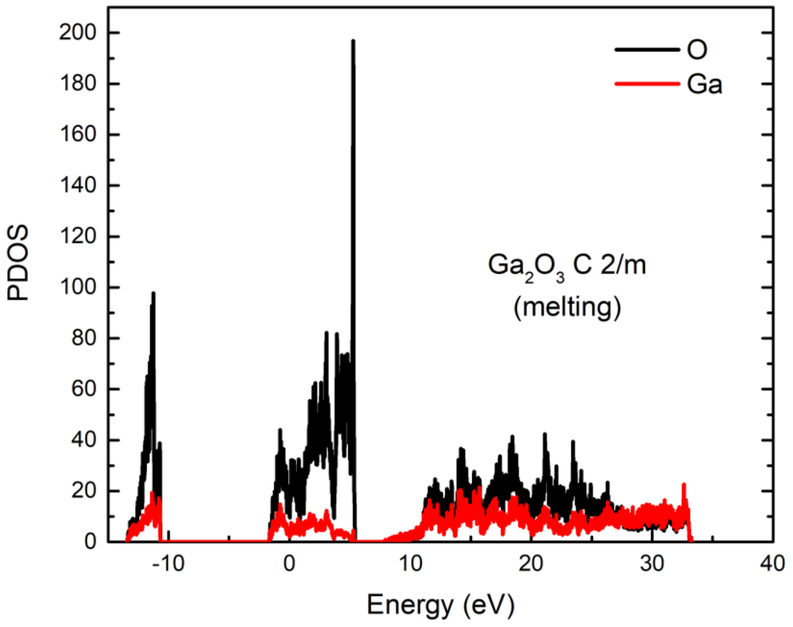

Figure 2.1.5. Total contribution of O and Ga atoms to electronic DOS of C2/m phase of Ga_2O_3.

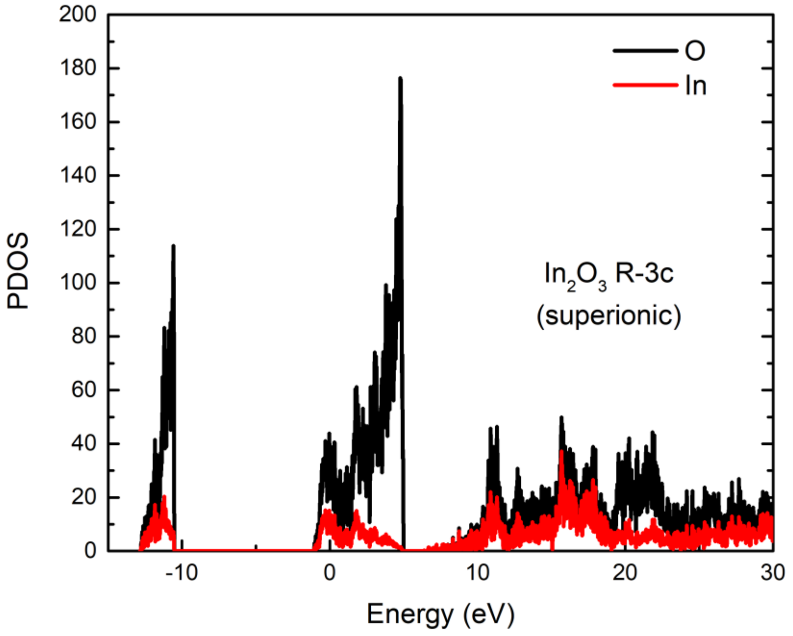

Figure 2.1.6. Total contribution of O and In atoms to electronic DOS of R-3c phase of In_2O_3.

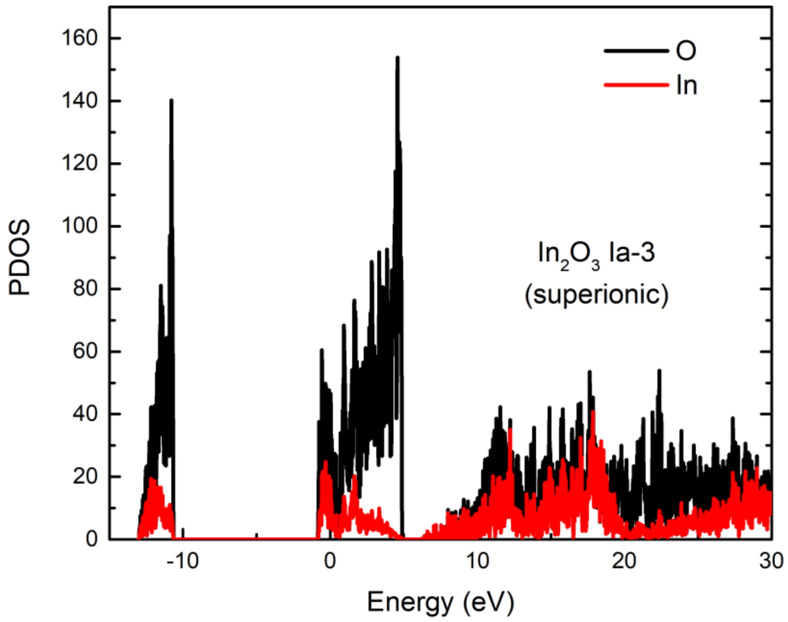

Figure 2.1.7. Total contribution of O and In atoms to electronic DOS of Ia-3 phase of In_2O_3.

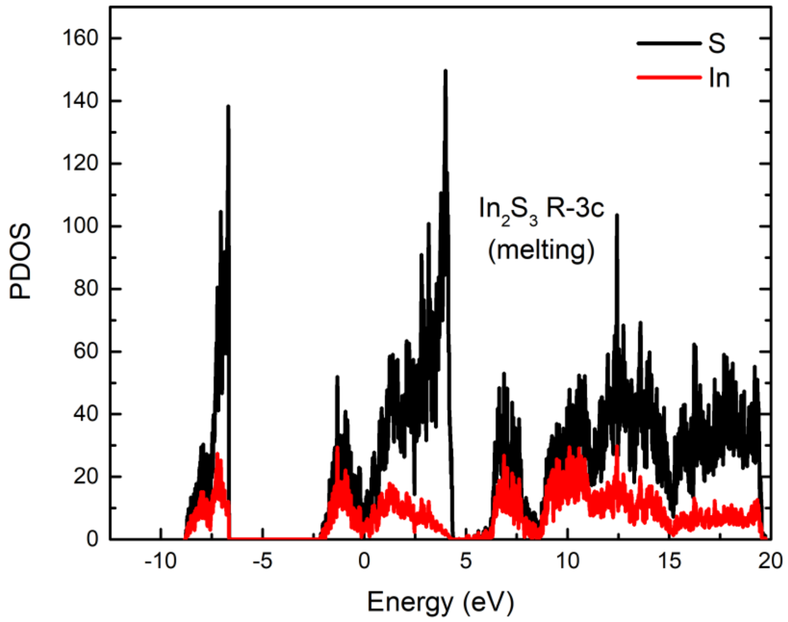

Figure 2.1.8. Total contribution of S and In atoms to electronic DOS of R-3c phase of In_2S_3.

2. Contribution of O orbitals to electronic DOS

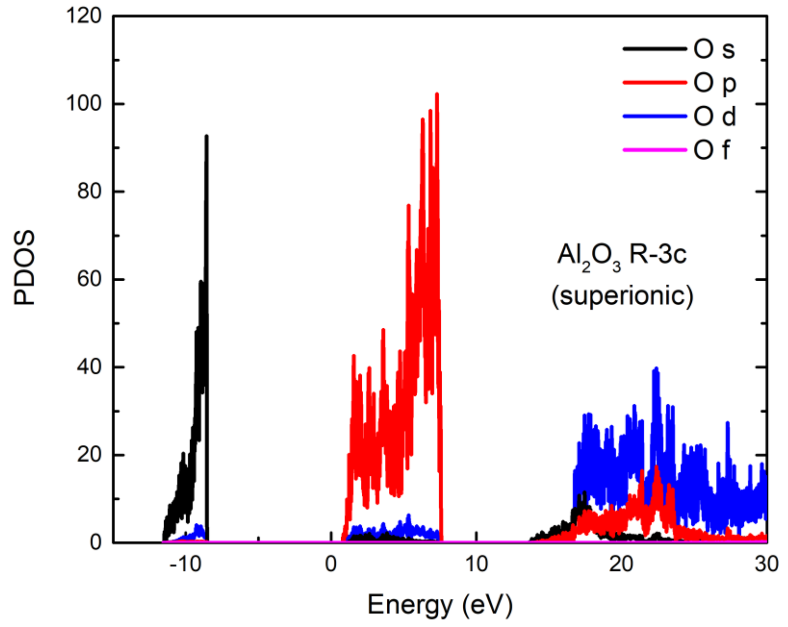

Figure 2.2.1. Contribution of O orbitals to electronic DOS of R-3c phase of Al_2O_3.

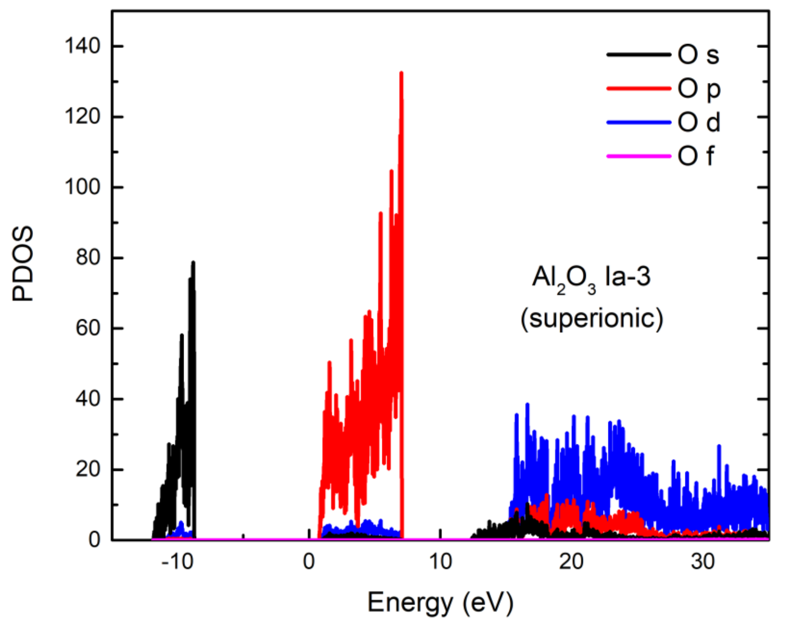

Figure 2.2.2. Contribution of O orbitals to electronic DOS of Ia-3 phase of Al_2O_3.

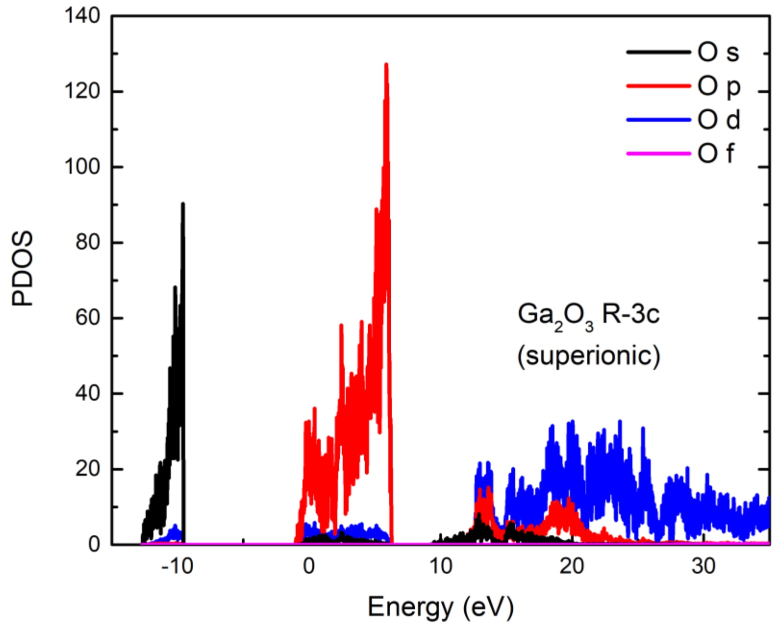

Figure 2.2.3. Contribution of O orbitals to electronic DOS of R-3c phase of Ga_2O_3.

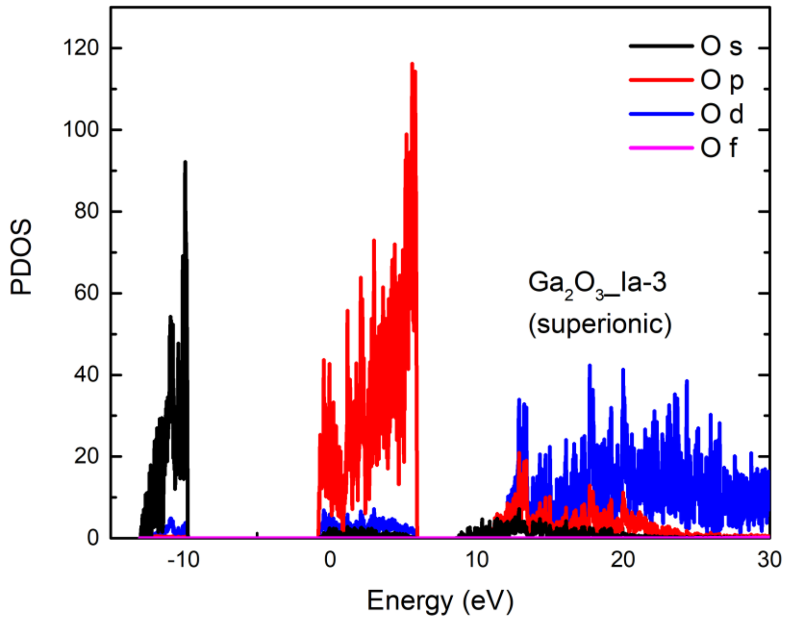

Figure 2.2.4. Contribution of O orbitals to electronic DOS of Ia-3 phase of Ga_2O_3.

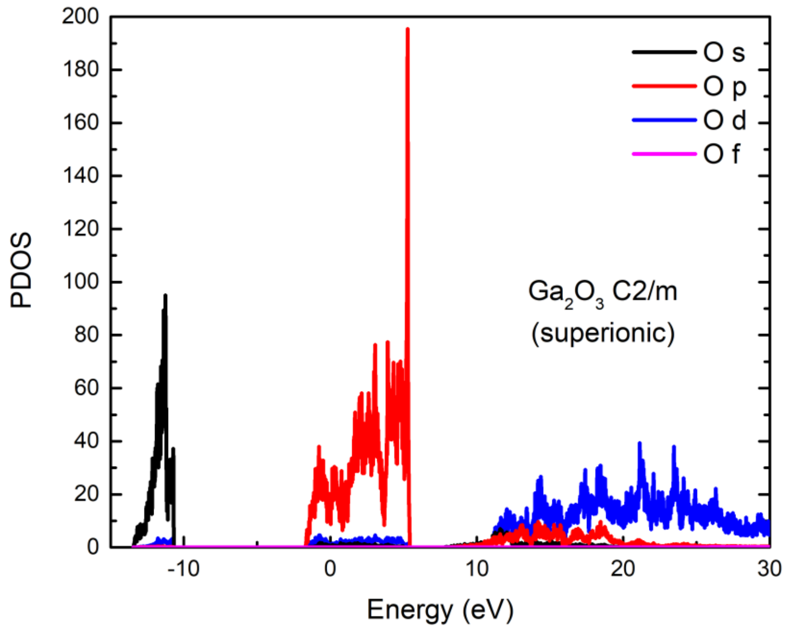

Figure 2.2.5. Contribution of O orbitals to electronic DOS of C2/m phase of Ga_2O_3.

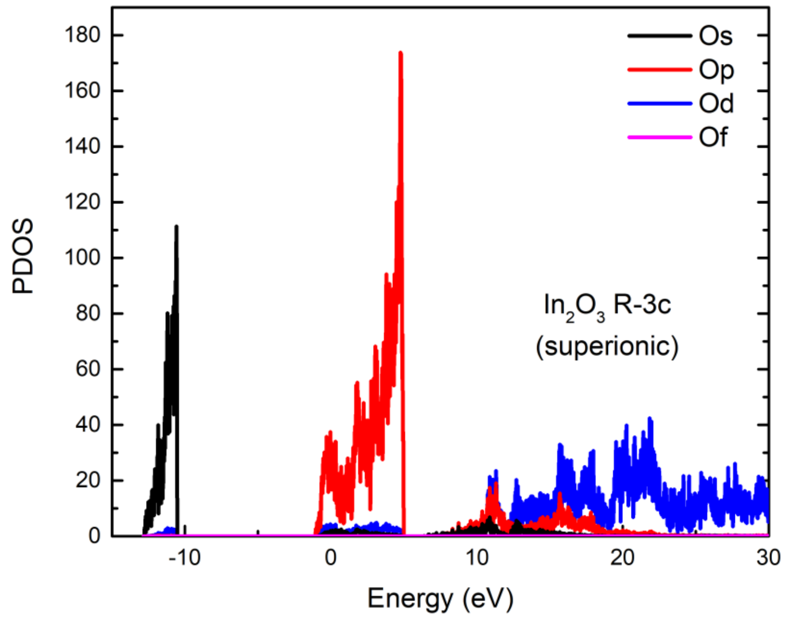

Figure 2.2.6. Contribution of O orbitals to electronic DOS of R-3c phase of In_2O_3.

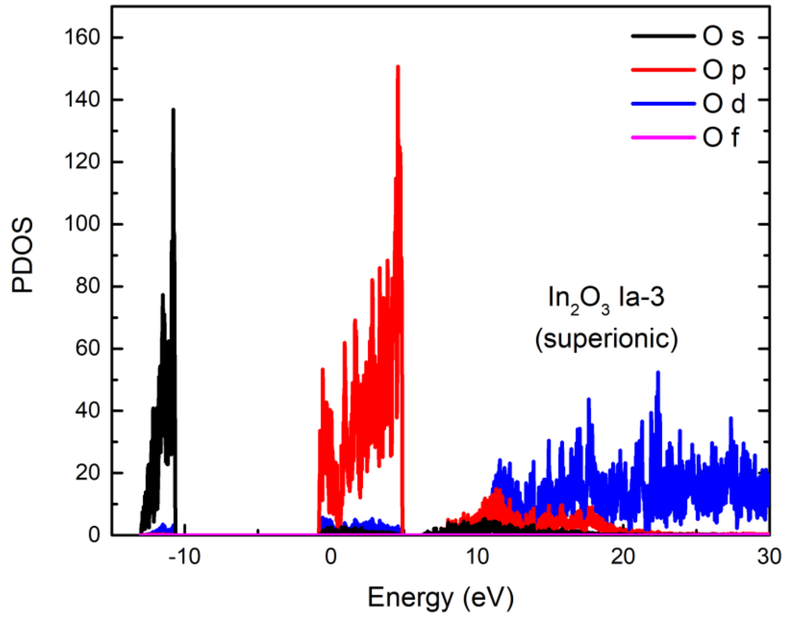

Figure 2.2.7. Contribution of O orbitals to electronic DOS of Ia-3 phase of In_2O_3.

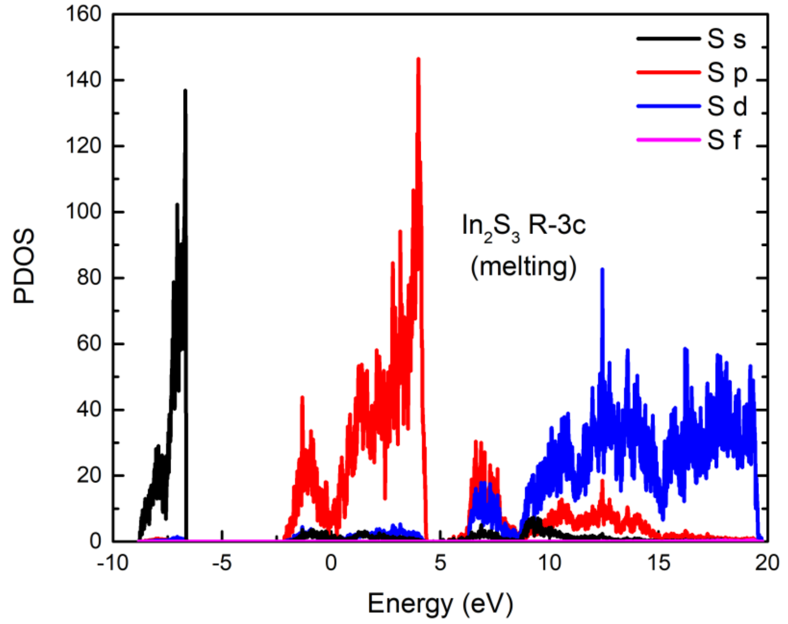

Figure 2.2.8. Contribution of S orbitals to electronic DOS of R-3c phase of In_2S_3.

3. Contribution of Me orbitals to electronic DOS

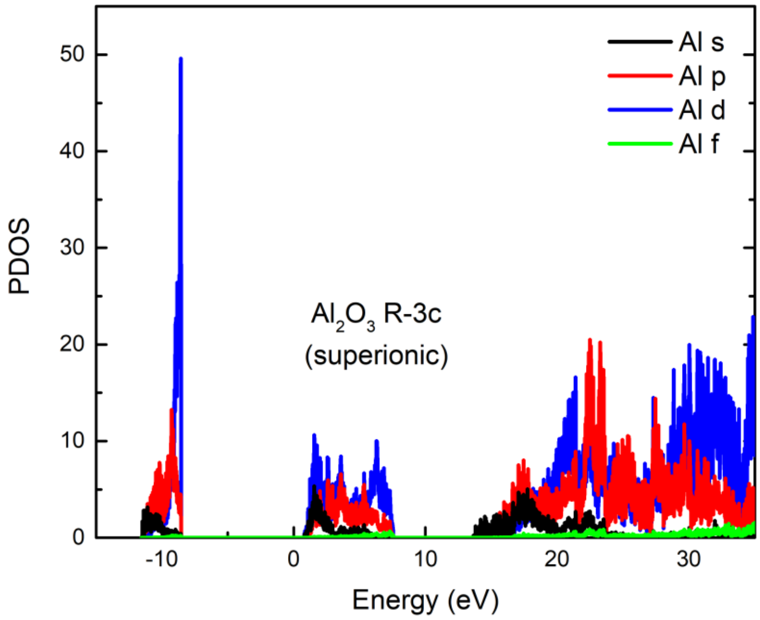

Figure 2.3.1. Contribution of Al orbitals to electronic DOS of R-3c phase of Al_2O_3.

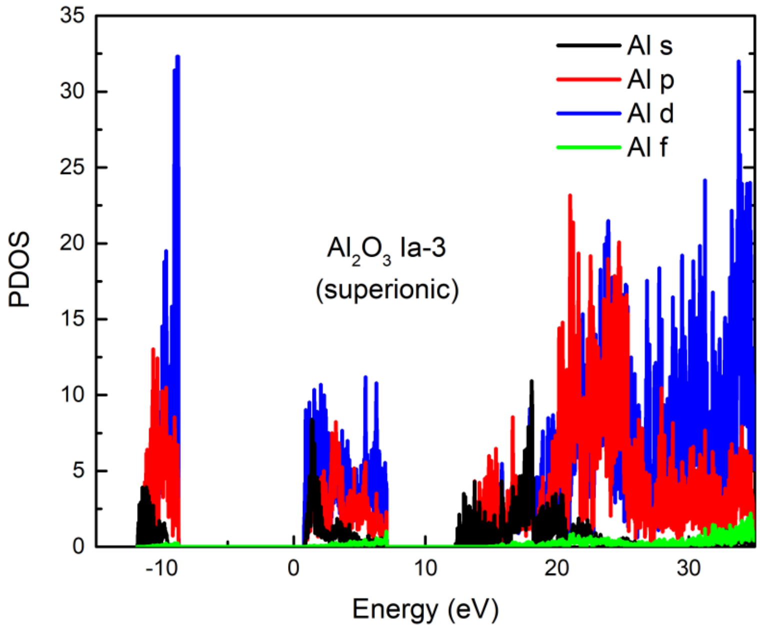

Figure 2.3.2. Contribution of Al orbitals to electronic DOS of Ia-3 phase of Al_2O_3.

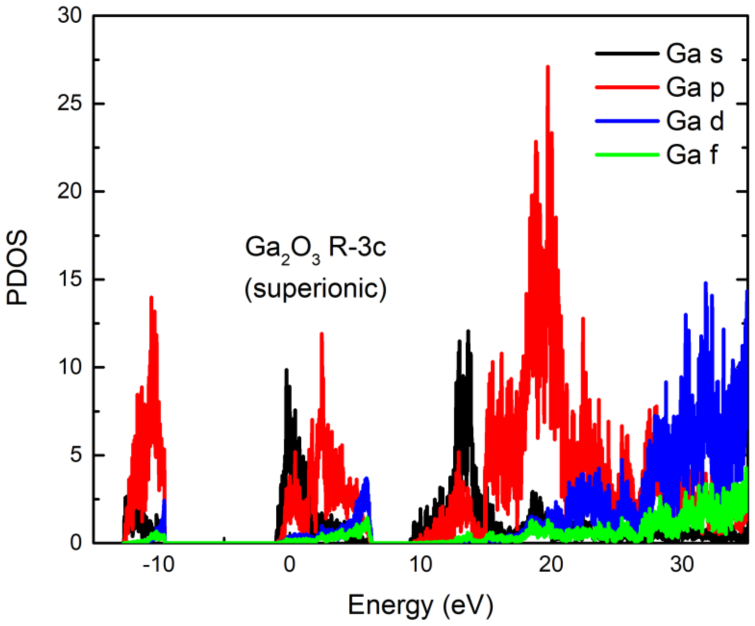

Figure 2.3.3. Contribution of Ga orbitals to electronic DOS of R-3c phase of Ga_2O_3.

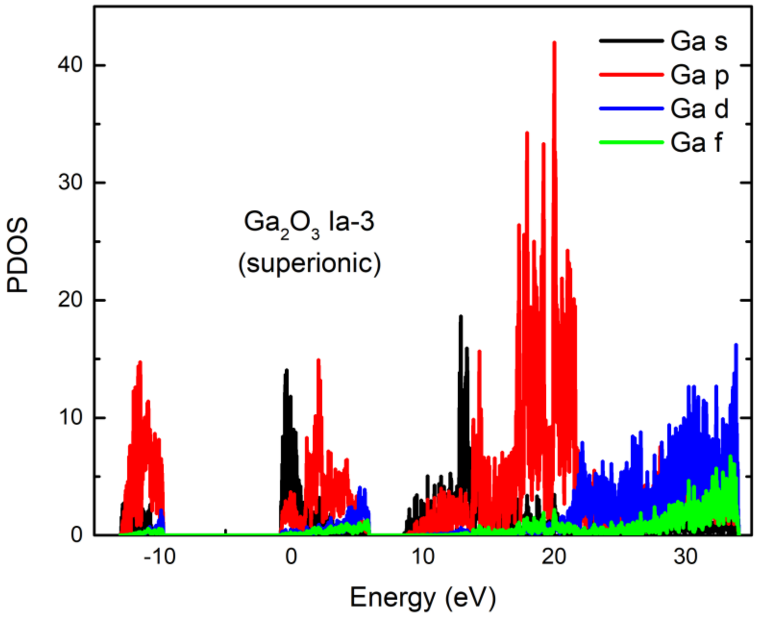

Figure 2.3.4. Contribution of Ga orbitals to electronic DOS of Ia-3 phase of Ga_2O_3.

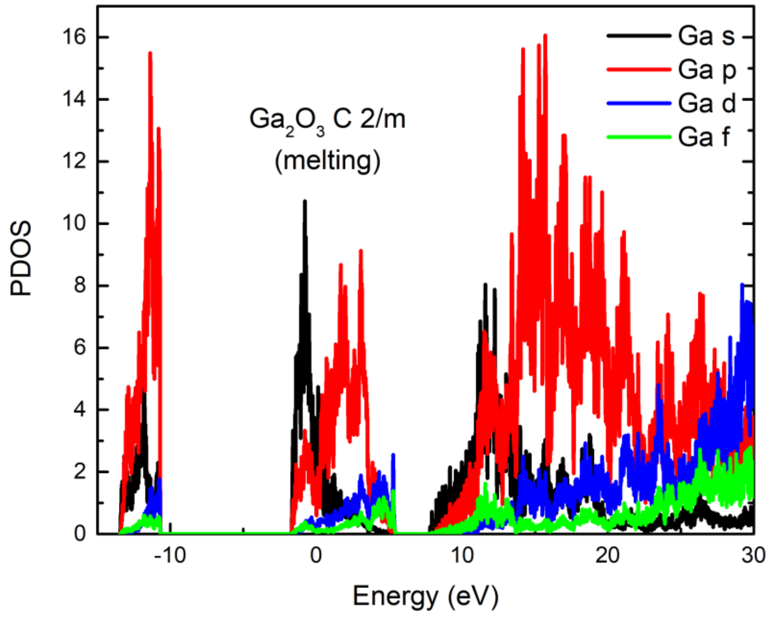

Figure 2.3.5. Contribution of Ga orbitals to electronic DOS of C2/m phase of Ga_2O_3.

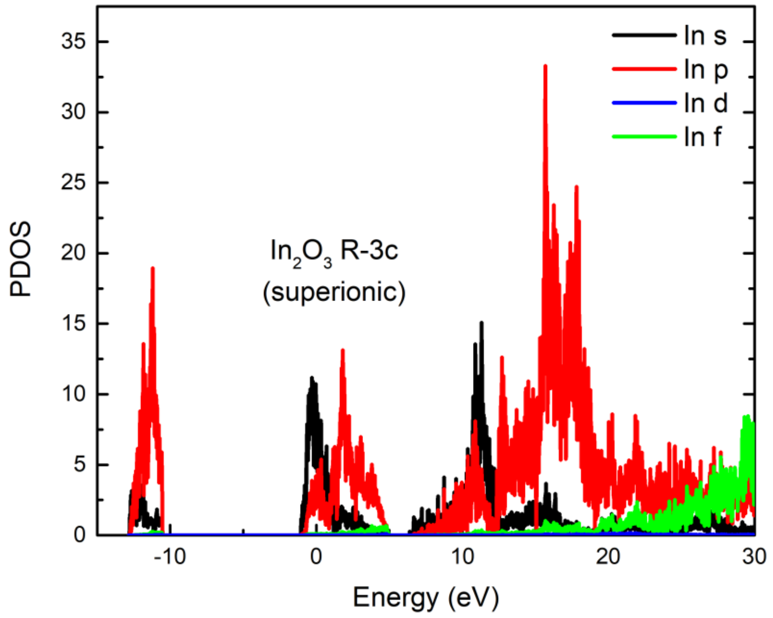

Figure 2.3.6. Contribution of In orbitals to electronic DOS of R-3c phase of In_2O_3.

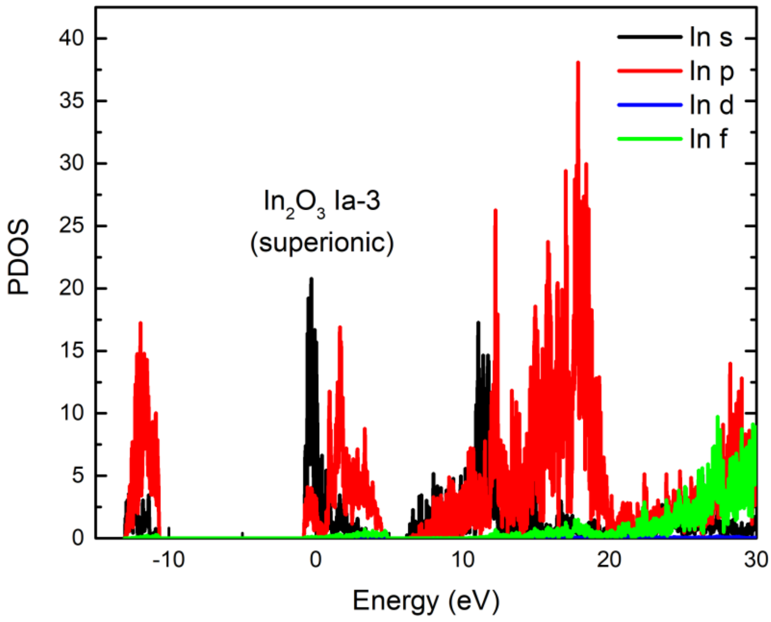

Figure 2.3.7. Contribution of In orbitals to electronic DOS of Ia-3 phase of In_2O_3.

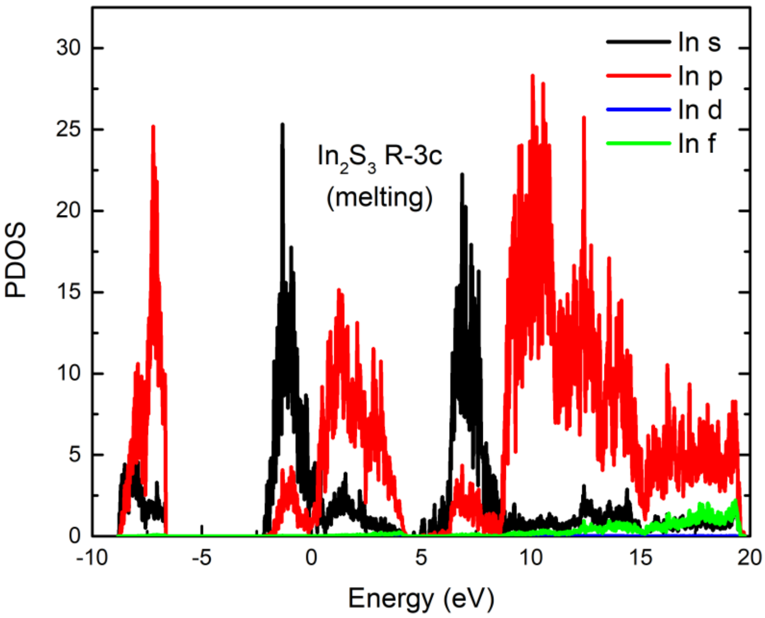

Figure 2.3.8. Contribution of In orbitals to electronic DOS of R-3c phase of In_2S_3.
